# Estimates for iterated commutators of multilinear square fucntions with Dini-type kernels

**DOI:** 10.1186/s13660-018-1778-8

**Published:** 2018-07-25

**Authors:** Zengyan Si, Qingying Xue

**Affiliations:** 1 School of Mathematics and Information Science, Henan Polytechnic University, Jiaozuo, People’s Republic of China; 2 School of Mathematical Sciences, Beijing Normal University, Beijing, People’s Republic of China; 3 Laboratory of Mathematics and Complex Systems, Ministry of Education, Beijing, People’s Republic of China

**Keywords:** Multilinear square fucntions, Triebel–Lizorkin spaces, Lipschitz spaces, Iterated commutators

## Abstract

Let $T_{\Pi\vec {b}}$ be the commutator generated by a multilinear square function and Lipschitz functions with kernel satisfying Dini-type condition. We show that $T_{\Pi\vec {b}}$ is bounded from product Lebesgue spaces into Lebesgue spaces, Lipschitz spaces, and Triebel–Lizorkin spaces.

## Introduction

Let $A(x)$ be an elliptic $n\times n$ matrix with complex-valued entries that are merely bounded and measurable, and let $T=\operatorname{div}(A(x)\nabla)$. The well-known problem of Kato is to show the boundedness of $T^{1/ 2}$ from the Sobolev space $H^{1}(\mathbb{R}^{n})$ to $L^{2}(\mathbb{R}^{n})$. Fabes et al. [6] studied a family of multilinear square functions and applied it to the Kato problem. In fact, they obtained a collection of multilinear Littlewood–Paley estimates and then applied them to two problems in partial differential equations. The first problem is the estimation of the square root of an elliptic operator in divergence form, and the second is the estimation of solutions to the Cauchy problem for nondivergence-form parabolic equations. Such a square function has important applications in PDEs and other fields’ we refer to [1–7, 9, 10, 13, 14, 17–19] and the references therein. We now give the definition of the multilinear square function of type $\omega(t)$.

Suppose that $\omega(t):[0,\infty)\mapsto[0,\infty)$ is a nondecreasing function with $0<\omega(1)<\infty$. For $a>0$, we say that *ω*∈ $\operatorname{Dini} (a)$ if

$$|\omega|_{\operatorname{Dini}(a)} = \int_{0}^{1} \omega^{a}(t) \frac{dt}{t}< \infty. $$

Let $K_{t}(x,y_{1},\ldots, y_{m})$ be a locally integrable function defined away from the diagonal $x=y_{1}=\cdots=y_{m}$ in $(\mathbb{R}^{n})^{m+1}$. We say that $K_{t}(x,y_{1},\ldots, y_{m})$ is a kernel of type $\omega(t)$ if there is a positive constant *A* such that the following conditions hold.

Size condition:

1.1 $$ \biggl( \int_{0}^{\infty}\bigl\vert K_{t}(x,y_{1}, \ldots,y_{m}) \bigr\vert ^{2} \frac{dt}{t} \biggr)^{\frac{1}{2}}\leq\frac{A}{(\sum_{j=1}^{m}|x-y_{j}|)^{mn}}. $$

Smoothness condition:

1.2 $$\begin{aligned} & \biggl( \int_{0}^{\infty}\bigl\vert K_{t}(z,y_{1}, \ldots,y_{m})-K_{t}(x,y_{1},\ldots ,y_{m}) \bigr\vert ^{2} \frac{dt}{t} \biggr)^{\frac{1}{2}} \\ &\quad \le\frac{A}{(\sum_{j=1}^{m}|x-y_{j}|)^{mn}}\omega \biggl(\frac {|z-x|}{\sum_{j=1}^{m}|x-y_{j}|} \biggr) \end{aligned}$$

whenever $|z-x|\leq\frac{1}{2} \max_{j=1}^{m}{|x-y_{j}|}$, and

1.3 $$\begin{aligned} & \biggl( \int_{0}^{\infty}\bigl\vert K_{t}(x,y_{1}, \ldots,y_{j},\ldots ,y_{m})-K_{t} \bigl(x,y_{1},\ldots,y'_{j}, \ldots,y_{m}\bigr) \bigr\vert ^{2} \frac{dt}{t} \biggr)^{\frac{1}{2}} \\ &\quad \leq\frac{A}{(\sum_{j=1}^{m}|x-y_{j}|)^{mn}}\omega \biggl(\frac {|y_{j}-y_{j}'|}{\sum_{j=1}^{m}|x-y_{j}|} \biggr) \end{aligned}$$

whenever $|y_{j}-y_{j}'|\leq\frac{1}{2} \max_{j=1}^{m}{|x-y_{j}|}$.

For any $x\notin\bigcap_{j=1}^{m} \mathtt{supp}\,\, f_{j}$ and $f_{j}\in C_{c}^{\infty}(\mathbb{R}^{n})$, we say *T* is a multilinear square function of type $\omega(t)$ if

1.4 $$ T(\vec{f}) (x)= \Biggl( \int_{0}^{\infty}\Biggl\vert \int_{(\mathbb {R}^{n})^{m}}K_{t}(x,y_{1}, \ldots,y_{m}) \prod_{j=1}^{m}f_{j}(y_{j}) \,dy_{1}\cdots \,dy_{m} \Biggr\vert ^{2} \frac{dt}{t} \Biggr)^{\frac{1}{2}}. $$

In this paper, we always assume that *T* can be extended to bounded operators from $L^{q_{1}}\times\cdots\times L^{q_{m}}$ to $L^{q}$ for some $1< q,q_{1},\ldots,q_{m}<\infty$ with $\frac{1}{q_{1}}+\cdots+\frac {1}{q_{m}}=\frac{1}{q}$.

### Remark 1.1

When $\omega(x)=x^{\gamma}$ for some $\gamma>0$, the boundedness of a multilinear square function was studied by Xue et al. [18].

### Definition 1.2

(Iterated commutators)

Given a collection of locally integrable functions $\vec{b}=(b_{1},\ldots,b_{m})$, the iterated commutator of a multilinear square function is defined by

1.5 $$\begin{aligned} &T_{\Pi\vec {b}}(\vec{f}) (x) \\ &\quad = \Biggl( \int_{0}^{\infty}\Biggl\vert \int_{(\mathbb{R}^{n})^{m}}\prod_{j=1}^{m} \bigl(b_{j}(x)-b_{j}(y_{j})\bigr)K(x,y_{1}, \ldots,y_{m})K_{t}(x,y_{1},\ldots,y_{m}) \\ &\qquad {}\times\prod_{j=1}^{m}f_{j}(y_{j}) \,dy_{1}\cdots \,dy_{m} \Biggr\vert ^{2} \frac{dt}{t} \Biggr)^{\frac{1}{2}}. \end{aligned}$$

### Definition 1.3

(Commutators in the *j*th entry)

Given a collection of locally integrable functions $\vec{b}=(b_{1},\ldots,b_{m})$, we define the commutator of a multilinear square function *T* as

$$[\vec{b},T](\vec{f})=T_{\vec{b}}(f_{1},\ldots,f_{m})= \sum_{j=1}^{m}T_{\vec{b}}^{j}( \vec{f}), $$

where each term is the commutator of $b_{j}$, and *T* in the *j*th entry of *T*, that is,

$$T_{\vec{b}}^{j}(\vec{f})=b_{j}T(f_{1}, \ldots,f_{j},\ldots,f_{m})-T(f_{1},\ldots ,b_{j}f_{j},\ldots,f_{m}). $$

For the commutators generated by the multilinear Calderón–Zygmud-type singular integrals and Lipschitz functions with the kernel of standard estimates, Wang and Xu [16] and Mo and Lu [11] obtained the boundedness from a product of Lebesgue spaces to the Lebesgue space, to the homogenous Triebel–Lizorkin space, and to Lipschitz spaces, respectively. Motivated by these results, we study the boundedness of commutators generated by the multilinear square functions and Lipschitz functions. The main results of this paper are as follows.

### Theorem 1.1

*Let* *T* *be a multilinear square function of type* $\omega(t)$ *with* $\omega\in\operatorname{Dini}(1)$. *Suppose* $b_{j}\in\dot{\wedge}_{\beta_{j}}$ *with* $0 < \beta _{j} < 1$ *for* $j = 1, \ldots,m$ *and* $\beta= \beta_{1} + \cdots+ \beta_{m}$. *If* $1 < p_{1}, \ldots, p_{m} <\infty$, $0< q <\infty$, *and* $1/p_{j} > \beta _{j}/n$ *with* $1/q = 1/p_{1}+\cdots+1/p_{m}-\beta/n$, *then* $T_{\Pi\vec {b}}$ *can be extended to a bounded operator from* $L^{p_{1}}\times\cdots \times L^{p_{m}}$ *into* $L^{q}$.

### Theorem 1.2

*Let* *T* *be a multilinear square function of type* $\omega(t)$ *with* $\omega\in\operatorname{Dini}(1)$. *Suppose* $b_{j}\in\dot{\wedge}_{\beta_{j}}$ *with* $0 < \beta _{j} < 1$ *for* $j = 1, \ldots,m$ *and* $\beta= \beta_{1} + \cdots+ \beta_{m}$. *Let* $1 < p_{1}, \ldots, p_{m} <\infty$, $0<1/p_{j} < \beta_{j}/n$, *and* $0<\beta -n/ p<1$ *with* $1/p = 1/p_{1}+\cdots+1/p_{m}$. *If* *ω* *satisfies*

$$\int_{0}^{1}\frac{\omega(t)}{t^{1+\beta-n/ p}}\,dt< \infty, $$

*then* $T_{\Pi\vec {b}}$ *can be extended to a bounded operator from* $L^{p_{1}}\times\cdots \times L^{p_{m}}$ *into Lipschitz space* $\dot{\wedge}_{\beta-n/ p}$.

### Theorem 1.3

*Let* *T* *be a multilinear square function of type* $\omega(t)$ *with* $\omega\in\operatorname{Dini}(1)$. *Suppose* $b_{j}\in\dot{\wedge}_{\beta_{j}}$ *with* $0 < \beta _{j} < 1$ *for* $j = 1, \ldots,m$ *and* $\beta= \beta_{1} + \cdots+ \beta_{m}$. *If* $1 < p_{1}, \ldots, p_{m} <\infty$ *with* $1/p = 1/p_{1}+\cdots+1/p_{m}$ *and* *ω* *satisfies*

$$\int_{0}^{1}\frac{\omega(t)}{t^{1+\beta}}\,dt< \infty, $$

*then* $T_{\Pi\vec {b}}$ *can be extended to a bounded operator from* $L^{p_{1}}\times\cdots \times L^{p_{m}}$ *into the Triebel–Lizorkin space* $\dot{F}_{p}^{\beta,\infty}$.

The paper is organized as follows. Some definitions and preliminaries are given in Sect. 2. In Sect. 3, we focus ourselves on a key lemma, which will be used in the proof of Theorem 1.1. The proofs of Theorems 1.2 and 1.3 are given in Sect. 4.

## Preliminaries

### Definition 2.1

For $\delta>0$, $M_{\delta}$ is the maximal function defined by

$$M_{\delta}f(x)=M\bigl( \vert f \vert ^{\delta}\bigr)^{\frac{1}{\delta}}(x)= \biggl( \sup_{Q\ni x}\frac{1}{ \vert Q \vert } \int_{Q} \bigl\vert f(y) \bigr\vert ^{\delta}\,dy \biggr)^{\frac {1}{\delta}}. $$

In addition, $M^{\sharp}$ is the sharp maximal function of Feffeman and Stein,

$$M^{\sharp}f(x)=\sup_{Q\ni x} \inf_{c} \frac{1}{ \vert Q \vert } \int_{Q} \bigl\vert f(y)-c \bigr\vert \,dy \approx\sup _{B\ni x} \frac{1}{ \vert Q \vert } \int_{Q} \bigl\vert f(y)-f_{Q} \bigr\vert \,dy, $$

and

$$M^{\sharp}_{\delta}f(x)= M^{\sharp}\bigl(|f|^{\delta}\bigr)^{\frac{1}{\delta}}(x). $$

Given a locally integrable function *f*, for $0\leq\beta< n$, we define the fractional maximal function

$$M_{r,\beta}f(x)=\sup_{x\in B} \biggl( \frac{1}{ \vert B \vert ^{1-{\beta r}/{n}}} \int_{B} \bigl\vert f(y) \bigr\vert ^{r}\,dy \biggr)^{\frac{1}{r}},\quad r\geq1. $$

If $\beta=0$ and $r=1$, then $M_{0,1}f=Mf$ denotes the usual Hardy–Littlewood maximal function. When $\beta=0$, we denote $M_{r,\beta}$ simply by $M_{r}$.

Chanillo [1] proved that if $0 <\beta< n, 0 < r < p< n/\beta$, and $1/q = 1/p-\beta/n$, then

$$\|M_{r,\beta}\|_{q}\leq C \|f\|_{p}. $$

### Definition 2.2

([12])

For $\beta>0$, the homogenous Lipschitz space $\dot{\wedge}_{\beta}(\mathbb{R}^{n})$ is the space of functions *f* such that

$$\|f\|_{\dot{\wedge}_{\beta}(\mathbb{R}^{n})}=\sup_{x,h\in\mathbb {R}^{n},h\neq0}\frac{|\Delta_{h}^{[\beta]+1}f(x)|}{|h|^{\beta}} < \infty, $$

where $\Delta_{h}^{k}$ denotes the *k*th difference operator.

To prove our theorem, we need the following lemmas.

### Lemma 2.1

([12])

*Let* $b\in\dot{\wedge}_{\beta}$, $0<\beta<1$. *For any cubes* $Q'$, *Q* *in* $\mathbb{R}^{n}$ *such that* $Q'\subset Q$, *we have*

$$\vert b_{Q'}-b_{Q} \vert \leq C \|b \|_{\dot{\wedge}_{\beta}}|Q|^{\beta/ n}. $$

### Lemma 2.2

([12])

1. *For* $0 <\beta< 1$ *and* $1 \leq q <\infty$, *we have*
  $$\|f\|_{\dot{\wedge}_{\beta}}\approx\sup_{Q} \frac{1}{|Q|^{1+n/ \beta }} \int_{Q} |f-f_{Q}|\approx\sup _{Q} \frac{1}{|Q|^{n/ \beta}} \biggl( \int _{Q} |f-f_{Q}|^{q} \biggr)^{\frac{1}{q}}. $$
2. *For* $0 <\beta< 1$ *and* $1 \leq p<\infty$, *we have*
  $$\|f\|_{\dot{F}^{\beta,\infty}_{p}}\approx \biggl\Vert \sup_{Q} \frac {1}{|Q|^{1+n/ \beta}} \int_{Q} |f-f_{Q}| \biggr\Vert _{L^{p}}. $$

### Lemma 2.3

([15])

*Let* $\frac{1}{p}=\frac {1}{p_{1}}+\cdots+\frac{1}{p_{m}}$ *and* $\vec{\omega}\in A_{\vec{p}}$. *Let* *T* *be a multilinear square function of type* $\omega(t)$ *with* $\omega\in \operatorname{Dini}(1)$.

1. *If* $1< p_{1}, \ldots, p_{m}<\infty$, *then*
  $$\|T\vec{f}\|_{L^{p}(\nu_{\vec{\omega}})}\leq C\prod_{i=1}^{m} \|f_{i}\|_{L^{p_{i}}(\omega_{i})}. $$
2. *If* $1\leq p_{1}, \ldots, p_{m}<\infty$, *then*
  $$\|T\vec{f}\|_{L^{p,\infty}(\nu_{\vec{\omega}})}\leq C\prod_{i=1}^{m} \|f_{i}\|_{L^{p_{i}}(\omega_{i})}. $$

## Proof of Theorem 1.1

To prove Theorem 1.1, we need the following estimates for $T_{\Pi\vec {b}}$ and $T^{j}_{ \vec{b}}$. We just consider the case $m=2$ for simplicity; our method still holds for general *m* with little modifications.

### Lemma 3.1

*Let* $0 <\delta<\epsilon< 1/ 2$, *and let* *T* *be a bilinear square function of type* $\omega(t)$ *with* *ω*∈ $\operatorname{Dini}(1)$.

(i) *If* $b_{1}\in\dot{\wedge}_{\beta_{1}} $ *and* $b_{2}\in\dot{\wedge }_{\beta_{2}} $ *with* $0<\beta_{1}, \beta_{2}<1$ *such that* $\beta_{1}+\beta_{2}=\beta$, *then*
  3.1 $$\begin{aligned} M^{\sharp}_{\delta}T_{\Pi\vec{b}}(f_{1},f_{2}) (x) \leq& C \Biggl\{ \prod_{i=1}^{2} \|b_{i}\|_{\dot{\wedge}_{\beta_{i}}} M_{\epsilon ,\beta} \bigl(T(f_{1},f_{2}) \bigr) (x) \\ &{}+\|b_{1}\|_{\dot{\wedge}_{\beta_{1}}} M_{\epsilon,\beta_{1}} \bigl(T^{2}_{\vec {b}}(f_{1},f_{2}) \bigr) (x) \\ &{}+\|b_{2}\|_{\dot{\wedge}_{\beta_{1}}} M_{\epsilon,\beta_{2}} \bigl(T^{1}_{\vec {b}}(f_{1},f_{2}) \bigr) (x) \\ &{}+\prod_{i=1}^{2}\|b_{i} \|_{\dot{\wedge}_{\beta_{i}}}M_{1,\beta _{1}}(f_{1}) (x) M_{1,\beta_{2}}(f_{2}) (x) \Biggr\} . \end{aligned}$$
(ii) *If* $b_{j}\in\dot{\wedge}_{\beta}$, $j=1,2 $, *and* $0<\beta<1$, *then*
  3.2 $$ M^{\sharp}_{\delta}T^{j}_{\vec{b}}(f_{1},f_{2}) (x)\leq C\|b_{j}\|_{\dot{\wedge}_{\beta}} \bigl\{ M_{\epsilon,\beta} \bigl(T(f_{1},f_{2})\bigr) (x)+ M_{1,\beta}(f_{j}) (x)M (f_{k}) (x) \bigr\} , $$
  *where* $k\neq j$, $k=1,2$.

### Proof

Fix a point *x* and a cube $Q(x_{Q},l)$ containing *x* with side-length *l*, and set $Q^{*}=8\sqrt{n}Q=Q(x_{Q},8\sqrt{n}l)$. We split $f_{j}$ as $f_{j}=f^{0}_{j}+f^{\infty}_{j}$, where $f^{0}_{j}=f_{j}\chi_{Q^{*}}$ and $f^{\infty}_{j}=f_{j}\chi_{\mathbb{R}^{n} \setminus Q^{*}}$ for $j=1,2$. As is well known, to obtain (3.1), it suffices to show that

$$\begin{aligned} & \biggl(\frac{1}{|Q|} \int_{Q} \bigl\vert T_{\Pi\vec{b}}(f_{1},f_{2}) (z)- c \bigr\vert ^{\delta }\,dz \biggr)^{\frac{1}{\delta}} \\ &\quad \leq C \Biggl\{ \prod_{i=1}^{2} \|b_{i}\|_{\dot{\wedge}_{\beta_{i}}} M_{\epsilon,\beta} \bigl(T(f_{1},f_{2}) \bigr) (x)+\|b_{1}\|_{\dot{\wedge}_{\beta _{1}}} M_{\epsilon,\beta_{1}} \bigl(T^{2}_{\vec{b}}(f_{1},f_{2})\bigr) (x) \\ &\qquad {} +\|b_{2}\|_{\dot{\wedge}_{\beta_{1}}} M_{\epsilon,\beta_{2}} \bigl(T^{1}_{\vec{b}}(f_{1},f_{2})\bigr) (x)+ \prod_{i=1}^{2}\|b_{i} \|_{\dot{\wedge }_{\beta_{i}}}M_{1,\beta_{1}}(f_{1}) (x) M_{1,\beta_{2}}(f_{2}) (x) \Biggr\} \end{aligned}$$

for some constant *c* to be determined.

Let $\lambda_{1}=(b_{1})_{Q^{*}}$ and $\lambda_{2}=(b_{2})_{Q^{*}}$. The sublinearity of $T_{\Pi\vec{b}}$ leads to

$$\begin{aligned} & \bigl\vert T_{\Pi\vec{b}}(f_{1},f_{2}) (z)- c \bigr\vert \\ &\quad \leq \bigl\vert \bigl(b_{1}(z)-\lambda_{1}\bigr) \bigl(b_{2}(z)-\lambda _{2}\bigr)T(f_{1},f_{2}) (z) \bigr\vert + \bigl\vert \bigl(b_{1}(z)-\lambda_{1} \bigr)T^{2}_{\vec{b}}(f_{1},f_{2}) (z) \bigr\vert \\ &\qquad {} + \bigl\vert \bigl(b_{2}(z)-\lambda_{2} \bigr)T^{1}_{\vec{b}}(f_{1},f_{2}) (z) \bigr\vert + \bigl\vert T\bigl((b_{1}-\lambda _{1})f_{1}, (b_{2}-\lambda_{2})f_{2}\bigr) (z)- c \bigr\vert . \end{aligned}$$

Thus we have

$$\begin{aligned} & \biggl(\frac{1}{ \vert Q \vert } \int_{Q} \bigl\vert T_{\Pi\vec{b}}(f_{1},f_{2}) (z)- c \bigr\vert ^{\delta }\,dz \biggr)^{\frac{1}{\delta}} \\ &\quad \leq \biggl(\frac{1}{ \vert B \vert } \int_{Q} \bigl\vert \bigl(b_{1}(z)- \lambda_{1}\bigr) \bigl(b_{2}(z)-\lambda _{2} \bigr)T(f_{1},f_{2}) (z) \bigr\vert ^{\delta}\,dz \biggr)^{\frac{1}{\delta}} \\ &\qquad {} + \biggl(\frac{1}{ \vert Q \vert } \int_{Q} \bigl\vert \bigl(b_{1}(z)- \lambda_{1}\bigr)T^{2}_{\vec {b}}(f_{1},f_{2}) (z) \bigr\vert ^{\delta}\,dz \biggr)^{\frac{1}{\delta}} \\ &\qquad {} + \biggl(\frac{1}{ \vert Q \vert } \int_{Q} \bigl\vert \bigl(b_{2}(z)- \lambda_{2}\bigr)T^{1}_{\vec {b}}(f_{1},f_{2}) (z) \bigr\vert ^{\delta}\,dz \biggr)^{\frac{1}{\delta}} \\ &\qquad {} + \biggl(\frac{1}{ \vert Q \vert } \int_{Q} \bigl\vert T\bigl((b_{1}- \lambda_{1})f_{1}, (b_{2}-\lambda_{2})f_{2} \bigr) (z)- c \bigr\vert ^{\delta}\,dz \biggr)^{\frac{1}{\delta}} \\ &\quad \doteq T_{1}+T_{2}+T_{3}+T_{4}. \end{aligned}$$

We now observe the elementary inequality

$$\bigl\vert b(z)-b_{Q} \bigr\vert \leq C|Q|^{\beta/ n}\|b \|_{\dot{\wedge}_{\beta}} $$

which follows from the fact $z\in Q$ and $b\in\dot{\wedge}_{\beta}$. From Hölder’s inequality and the assumption $\beta_{1}+\beta _{2}=\beta$, for $0 <\delta<\epsilon< 1/ 2$, we have

$$\begin{aligned} T_{1} &\leq \prod_{i=1}^{2} \|b_{i}\|_{\dot{\wedge}_{\beta_{i}}} \biggl(\frac {1}{|Q|^{1-\frac{\delta\beta}{n}}} \int_{Q} \bigl\vert T(f_{1},f_{2}) (z) \bigr\vert ^{\delta }\,dz \biggr)^{\frac{1}{\delta}} \\ &\leq\prod_{i=1}^{2}\|b_{i} \|_{\dot{\wedge}_{\beta_{i}}} \biggl(\frac {1}{|Q|^{1-\frac{\epsilon\beta}{n}}} \int_{Q} \bigl\vert T(f_{1},f_{2}) (z) \bigr\vert ^{\epsilon }\,dz \biggr)^{\frac{1}{\epsilon}} \\ &\leq C \prod_{i=1}^{2}\|b_{i} \|_{\dot{\wedge}_{\beta_{i}}} M_{\epsilon ,\beta} \bigl(T(f_{1},f_{2}) \bigr) (x). \end{aligned}$$

Similarly, we have

$$ T_{2}\leq C \|b_{1}\|_{\dot{\wedge}_{\beta_{1}}} M_{\epsilon,\beta_{1}} \bigl(T^{2}_{\vec{b}}(f_{1},f_{2}) \bigr) (x) $$

and

$$ T_{3}\leq \|b_{2}\|_{\dot{\wedge}_{\beta_{2}}} M_{\epsilon,\beta_{2}} \bigl(T^{1}_{\vec {b}}(f_{1},f_{2}) \bigr) (x). $$

Now we deal with $T_{4}$. Set $c= T((b_{1}-\lambda_{1})f_{1}^{\infty },(b_{2}-\lambda_{2})f_{2}^{\infty})(x)$. We may bound $T_{4}$ as

$$T_{4}\leq T_{41}+T_{42}+T_{43}+T_{44}, $$

where

$$\begin{aligned}& T_{41}= \biggl(\frac{1}{ \vert Q \vert } \int_{Q} \bigl\vert T\bigl((b_{1}- \lambda_{1})f_{1}^{0}, (b_{2}- \lambda_{2})f_{2}^{0}\bigr) (z) \bigr\vert ^{\delta}\,dx \biggr)^{\frac{1}{\delta}}, \\& T_{42}= \biggl(\frac{1}{ \vert Q \vert } \int_{Q} \bigl\vert T\bigl((b_{1}- \lambda_{1})f_{1}^{\infty}, (b_{2}- \lambda_{2})f_{2}^{0}\bigr) (z) \bigr\vert ^{\delta}\,dz \biggr)^{\frac{1}{\delta}}, \\& T_{43}= \biggl(\frac{1}{ \vert Q \vert } \int_{Q} \bigl\vert T\bigl((b_{1}- \lambda_{1})f_{1}^{0}, (b_{2}- \lambda_{2})f_{2}^{\infty}\bigr) (z) \bigr\vert ^{\delta}\,dz \biggr)^{\frac{1}{\delta}}, \end{aligned}$$

and

$$T_{44}= \biggl(\frac{1}{ \vert Q \vert } \int_{Q} \bigl\vert T\bigl((b_{1}- \lambda_{1})f_{1}^{\infty}, (b_{2}- \lambda_{2})f_{2}^{\infty}\bigr) (z)- T \bigl((b_{1}-\lambda_{1})f_{1}^{\infty}, (b_{2}-\lambda_{2})f_{2}^{\infty }\bigr) (x) \bigr\vert ^{\delta}\,dz \biggr)^{\frac{1}{\delta}}. $$

For $T_{41}$, by Kolmogorov’s inequality and Lemma 2.3 we get

$$\begin{aligned} T_{41}& \leq C \bigl\Vert T\bigl((b_{1}- \lambda_{1})f_{1}^{0}, (b_{2}-\lambda _{2})f_{2}^{0}\bigr) \bigr\Vert _{L^{1/2,\infty}(B, \frac{dx}{|Q|})} \\ &\leq\frac{C}{ \vert Q \vert } \int_{Q} \bigl\vert (b_{1}-\lambda_{1})f_{1}^{0}(z) \bigr\vert \,dz \frac {1}{ \vert Q \vert } \int_{Q} \bigl\vert (b_{2}-\lambda_{2})f_{2}^{0}(z) \bigr\vert \,dz \\ &\leq C\|b_{1}\|_{\dot{\wedge}_{\beta_{1}}}\|b_{2}\|_{\dot{\wedge }_{\beta_{2}}} M_{1,\beta_{1}}(f_{1}) (x) M_{1,\beta_{2}}(f_{2}) (x). \end{aligned}$$

For any $y\in\mathbb{R}^{n} \setminus Q^{*}$ and $b\in\dot{\wedge }_{\beta}$, there exists $Q'$ such that $Q^{*}\subset Q'$ and $|y-x_{Q}|\sim |Q'|^{1/ n}$. Then, from Lemma 2.1 we have

3.3 $$ \bigl\vert b(y)-b_{Q^{*}} \bigr\vert \leq \bigl\vert b(y)-b_{Q'} \bigr\vert +|b_{Q'}-b_{Q^{*}}|\leq C \|b\|_{\dot {\wedge}_{\beta}}|y-x_{Q}|^{\beta}. $$

For any $y_{2}\in(Q^{*})^{c}$ and $z\in Q$, we have $|z-y_{2}|\sim|y_{2}-x_{Q}|$. By Minkowski’s inequality and the size condition (1.1) we get

$$\begin{aligned} T_{43}&\leq \biggl(\frac{1}{|Q|} \int_{Q} \bigl\vert T\bigl((b_{1}- \lambda_{1})f_{1}^{0}, (b_{2}-\lambda _{2})f_{2}^{\infty}\bigr) (z) \bigr\vert ^{\delta}\,dz \biggr)^{\frac{1}{\delta}} \\ &\leq C \biggl(\frac{1}{|Q|} \int_{Q} \biggl\vert \int_{\mathbb{R}^{nm}} \biggl( \int_{0}^{\infty}\bigl\vert K_{t}(z,y_{1},y_{2}) \bigr\vert ^{2}\frac{dt}{t} \biggr)^{1/ 2} \\ &\quad {} \times \bigl\vert \bigl(b_{1}(y_{1})- \lambda_{1}\bigr)f_{1}^{0}(y_{1}) \bigr\vert \bigl\vert \bigl(b_{2}(y_{2})-\lambda _{2} \bigr)f_{2}^{\infty}(y_{2}) \bigr\vert \,dy_{1}\,dy_{2} \biggr\vert ^{\delta}\,dz \biggr)^{\frac{1}{\delta}} \\ &\leq C \biggl(\frac{1}{|Q|} \int_{Q} \biggl\vert \int_{Q^{*}} \int_{\mathbb {R}^{n}\setminus Q^{*}}\frac{|(b_{1}(y_{1})-\lambda_{1})f_{1}^{0}(y_{1})||(b_{2}(y_{2})-\lambda _{2})f_{2}^{\infty}(y_{2})|\,dy_{1}\,dy_{2}}{(|z-y_{1}|+|z-y_{2}|)^{2n}} \biggr\vert ^{\delta}\,dz \biggr)^{\frac {1}{\delta}} \\ &\leq C\|b_{1}\|_{\dot{\wedge}_{\beta_{1}}}M_{1,\beta_{1}}(f_{1}) (x)|Q| \sum_{k=1}^{\infty}\int_{2^{k+3 }\sqrt{n}Q\setminus2^{k+2}\sqrt {n}Q}\frac{|f_{2}(y_{2})(b_{2}(y_{2})-\lambda_{2})|\,dy_{2}}{|y_{2}-x_{Q}|^{2n}} \\ &\leq C\|b_{1}\|_{\dot{\wedge}_{\beta_{1}}}M_{1,\beta_{1}}(f_{1}) (x)|Q| \sum_{k=1}^{\infty}\frac{1}{|2^{k+3 }\sqrt{n}Q|^{1-\beta_{2}}} \int _{2^{k+3 }\sqrt{n}Q }\bigl|f_{2}(y_{2})\bigr|\,dy_{2} \\ &\leq C\|b_{1}\|_{\dot{\wedge}_{\beta_{1}}}\|b_{2}\|_{\dot{\wedge }_{\beta_{2}}}M_{1,\beta_{1}}(f_{1}) (x) M_{1,\beta_{2}}(f_{2}) (x)\sum_{k=1}^{\infty}2^{-k} \\ &\leq C\|b_{1}\|_{\dot{\wedge}_{\beta_{1}}}\|b_{2}\|_{\dot{\wedge }_{\beta_{2}}}M_{1,\beta_{1}}(f_{1}) (x) M_{1,\beta_{2}}(f_{2}) (x). \end{aligned}$$

By using the same technique we get $T_{42}\leq C\|b_{1}\|_{\dot{\wedge }_{\beta_{1}}} \|b_{2}\|_{\dot{\wedge}_{\beta_{2}}}M_{1,\beta _{1}}(f_{1})(x)M_{1,\beta_{2}}(f_{2})(x)$.

To estimate $T_{44}$, we use Minkowski’s inequality and (1.2) and (3.3). Since $(\mathbb{R}^{n} \setminus Q^{*})^{2}\subseteq \mathbb{R}^{2n}\setminus(Q^{*})^{2}\subseteq\bigcup_{k=1}^{\infty}(2^{k+3 }\sqrt{n}Q)^{2}\setminus(2^{k+2 }\sqrt{n}Q)^{2}$, we deduce that

$$\begin{aligned} T_{44}&\leq\Biggl(\frac{1}{ \vert Q|} \int_{Q} \Biggl\vert \int_{\mathbb{R}^{nm}}\biggl( \int_{0}^{\infty}\bigl\vert K_{t}(z,\vec {y})-K_{t}(x,\vec{y}) \bigr\vert ^{2}\frac{dt}{t} \biggr)^{1/ 2} \\ &\quad {}\times\prod_{i=1}^{2} \bigl\vert \bigl(b_{i}(y_{i})-\lambda_{i} \bigr)f_{i}^{\infty}(y_{i}) \bigr\vert \, d \vec{y} \Biggr\vert ^{\delta}\,dz \Biggr)^{\frac{1}{\delta}} \\ &\leq C\Biggl(\frac{1}{|Q|} \int_{Q} \Biggl\vert \int_{(\mathbb {R}^{n}\setminus Q^{*})^{2}}\frac{1}{(|x-y_{1}|+|x-y_{2}|)^{2n}}\omega\biggl(\frac {|z-x_{Q}|}{|x-y_{1}|+|x-y_{2}|} \biggr) \\ &\quad {} \times\prod_{i=1}^{2} \bigl\vert \bigl(b_{i}(y_{i})-\lambda_{i} \bigr)f_{i}^{\infty}(y_{i})\bigr|\,d \vec{y}\Biggr|^{\delta}\,dz\Biggr)^{\frac{1}{\delta}} \\ &\leq C\frac{1}{|Q|} \int_{Q}\sum_{k=1}^{\infty}\int_{(2^{k+3 }\sqrt {n}Q\setminus2^{k+2}\sqrt{n}Q)^{2}}\frac{1}{(|2^{k+3 }\sqrt {n}Q|)^{2}}\omega\bigl(2^{-k}\bigr) \\ &\quad {}\times\prod_{i=1}^{2} \bigl\vert \bigl(b_{i}(y_{i})-\lambda _{i} \bigr)f_{i}^{\infty}(y_{i}) \bigr\vert \, d \vec{y}\,dz \\ &\leq C\sum_{k=1}^{\infty}\frac{1}{(|2^{k+3 }\sqrt{n}Q|)^{2-\beta _{1}-\beta_{2}}} \int_{(2^{k+3 }\sqrt{n}Q\setminus2^{k+2}\sqrt {n}Q)^{2}}\omega\bigl(2^{-k}\bigr)\prod _{i=1}^{2} \bigl\vert f_{i}^{\infty}(y_{i}) \bigr\vert \, d\vec{y} \\ &\leq C\|b_{1}\|_{\dot{\wedge}_{\beta_{1}}}\|b_{2}\|_{\dot{\wedge }_{\beta_{2}}}M_{1,\beta_{1}}(f_{1}) (x) M_{1,\beta_{2}}(f_{2}) (x). \end{aligned}$$

Combing all our estimates together, we obtain (3.1).

Now we are in the position to prove (3.2). It is sufficient to consider the operator with only one symbol. Fix $b\in\dot{\wedge}_{\beta}$ and consider the operator

$$T_{b}(\vec{f}) (x)= \bigl\vert b(x)T(f_{1},f_{2}) (x)-T(bf_{1},f_{2}) (x) \bigr\vert . $$

We have to prove that

$$ M^{\sharp}_{\delta}T_{b}(f_{1},f_{2}) (x)\leq C\|b\|_{\dot{\wedge}_{\beta}} \bigl\{ M_{\epsilon,\beta} \bigl(T(f_{1},f_{2}) \bigr) (x)+ M_{1,\beta}(f_{1}) (x)M (f_{2}) (x) \bigr\} . $$

Let $\lambda=b_{Q^{*}}$. We can control $T_{b}(\vec{f})(x)$ as

$$T_{b}(\vec{f}) (x)\leq \bigl\vert \bigl(b(x)-\lambda\bigr) \bigr\vert T(f_{1},\ldots ,f_{m}) (x)+T\bigl((b- \lambda)f_{1},\ldots,f_{m}\bigr) (x). $$

Then, for any constant *c*, we obtain that

$$\begin{aligned} & \biggl(\frac{1}{|Q|} \int_{Q} \bigl\vert \bigl\vert T_{ b}(f_{1},f_{2}) (z) \bigr\vert ^{\delta}- \vert c|^{\delta}\bigr|\,dz \biggr)^{\frac{1}{\delta}} \\ &\quad \leq \biggl(\frac {1}{ \vert Q \vert } \int_{Q} \bigl\vert T_{ b}(f_{1},f_{2}) (z)- c \bigr\vert ^{\delta}\,dz \biggr)^{\frac {1}{\delta}} \\ &\quad \leq \biggl(\frac{1}{ \vert Q \vert } \int_{Q} \bigl\vert \bigl(b(z)-\lambda \bigr)T(f_{1},f_{2}) (z) \bigr\vert ^{\delta}\,dz \biggr)^{\frac{1}{\delta}} + \biggl(\frac{1}{ \vert Q \vert } \int_{Q} \bigl\vert T\bigl((b-\lambda)f_{1}, f_{2}\bigr) (z)- c \bigr\vert ^{\delta}\,dz \biggr)^{\frac{1}{\delta}} \\ &\quad =: (P_{1}+P_{2}). \end{aligned}$$

By Hölder’s inequality we get

$$\begin{aligned} P_{1}&\leq C\|Q\|_{\dot{\wedge}_{\beta}} \biggl(\frac{1}{|Q|^{1-\frac{\delta \beta}{n}}} \int_{Q} \bigl\vert T(f_{1},f_{2}) (z) \bigr\vert ^{\delta}\,dz \biggr)^{\frac {1}{\delta}} \\ &\leq C \|b\|_{\dot{\wedge}_{\beta}} M_{\epsilon,\beta} \bigl(T(f_{1},f_{2}) \bigr) (x). \end{aligned}$$

We bound the second part as

$$P_{2}\leq P_{21}+P_{22}+P_{23}+P_{24}, $$

where

$$\begin{aligned}& P_{21}= \biggl(\frac{1}{ \vert Q \vert } \int_{Q} \bigl\vert T\bigl((b-\lambda)f_{1}^{0}, f_{2}^{0}\bigr) (z) \bigr\vert ^{\delta}\,dx \biggr)^{\frac{1}{\delta}}, \\& P_{22}= \biggl(\frac{1}{ \vert Q \vert } \int_{Q} \bigl\vert T\bigl((b-\lambda)f_{1}^{0}, f_{2}^{\infty }\bigr) (z) \bigr\vert ^{\delta}\,dz \biggr)^{\frac{1}{\delta}}, \\& P_{23}= \biggl(\frac{1}{ \vert Q \vert } \int_{Q} \bigl\vert T\bigl((b-\lambda)f_{1}^{\infty}, f_{2}^{0}\bigr) (z) \bigr\vert ^{\delta}\,dz \biggr)^{\frac{1}{\delta}}, \end{aligned}$$

and

$$P_{24}= \biggl(\frac{1}{ \vert Q \vert } \int_{Q} \bigl\vert T\bigl((b-\lambda)f_{1}^{\infty}, f_{2}^{\infty}\bigr) (z)- T \bigl((b-\lambda)f_{1}^{\infty}, f_{2}^{\infty }\bigr) (x) \bigr\vert ^{\delta}\,dz \biggr)^{\frac{1}{\delta}}. $$

By Kolmogorov’s inequality and Lemma 2.3 we get

$$\begin{aligned} P_{21}& \leq C \bigl\Vert T\bigl((b-\lambda)f_{1}^{0}, f_{2}^{0}\bigr) \bigr\Vert _{L^{1/2,\infty}(B, \frac{dx}{|Q|})} \\ &\leq\frac{ C}{ \vert Q \vert } \int_{Q} \bigl\vert (b-\lambda)f_{1}^{0}(z) \bigr\vert \,dz \frac{1}{ \vert Q \vert } \int_{Q} \bigl\vert f_{2}^{0}(z) \bigr\vert \,dz \\ &\leq C \Vert b \Vert _{\dot{\wedge}_{\beta}} \bigl\vert Q^{*} \bigr\vert ^{\beta/ n}\frac{1}{ \vert Q \vert } \int_{Q} \bigl\vert f_{1}^{0}(z) \bigr\vert \,dz \frac{1}{ \vert Q \vert } \int_{Q} \bigl\vert f_{2}^{0}(z) \bigr\vert \,dz \\ &\leq C \Vert b \Vert _{\dot{\wedge}_{\beta}}M_{1,\beta}(f_{1}) (x)M (f_{2}) (x). \end{aligned}$$

By using the Minkowski inequality and (1.1) and (3.3) we obtain that

$$\begin{aligned} P_{22}&= \biggl(\frac{1}{ \vert Q \vert } \int_{Q} \bigl\vert T\bigl((b-\lambda)f_{1}^{0}, f_{2}^{\infty}\bigr) (z) \bigr\vert ^{\delta}\,dz \biggr)^{\frac{1}{\delta}} \\ &\leq C\frac{1}{ \vert Q \vert } \int_{Q} \bigl\vert T\bigl((b-\lambda)f_{1}^{0}, f_{2}^{\infty }\bigr) (z) \bigr\vert \,dz \\ &\leq C\frac{1}{ \vert Q \vert } \int_{Q} \int_{Q^{*}} \int_{(Q^{*})^{c}}\biggl( \int _{0}^{\infty}\bigl\vert K_{t}(z,y_{1},y_{2}) \bigr\vert ^{2}\frac{dt}{t}\biggr)^{1/ 2} \\ &\quad {}\times\bigl\vert \bigl(b(y_{1})-\lambda\bigr)f_{1}(y_{1}) \bigr\vert \bigl\vert f_{2}(y_{2}) \bigr\vert \,dy_{2} \,dy_{1} \,dz \\ &\leq C\frac{1}{ \vert Q \vert } \int_{Q} \int_{Q^{*}} \int_{(Q^{*})^{c}}\frac {1}{( \vert z-y_{1} \vert + \vert z-y_{2} \vert )^{2n}} \bigl\vert \bigl(b(y_{1})- \lambda\bigr)f_{1}(y_{1}) \bigr\vert \bigl\vert f_{2}(y_{2}) \bigr\vert \,dy_{2} \,dy_{1} \,dz \\ &\leq C \int_{Q^{*}} \bigl\vert \bigl(b(y_{1})-\lambda \bigr)f_{1}(y_{1}) \bigr\vert \,dy_{1} \int_{\mathbb {R}^{n}\setminus Q^{*}}\frac{ \vert f_{2}(y_{2}) \vert \,dy_{2}}{ \vert z-y_{2} \vert ^{2n}} \\ &\leq C\|b\|_{\dot{\wedge}_{\beta}} \int_{Q^{*}} \vert y_{1}-x_{Q} \vert ^{\beta}\bigl\vert f_{1}(y_{1}) \bigr\vert \,dy_{1} \sum_{k=1}^{\infty}\int_{2^{k+1}Q^{*}\setminus2^{k}Q^{*}}\frac { \vert f_{2}(y_{2}) \vert \,dy_{2}}{ \vert z-y_{2} \vert ^{2n}} \\ &\leq C\|b\|_{\dot{\wedge}_{\beta}}\sum_{k=1}^{\infty}\bigl\vert 2^{k}Q^{*} \bigr\vert ^{-2} \bigl\vert Q^{*} \bigr\vert \int_{2^{k+1}Q^{*}\setminus 2^{k}Q^{*}} \bigl\vert f_{2}(y_{2}) \bigr\vert \,dy_{2}M_{1,\beta}(f_{1}) (x) \\ &\leq C\|b\|_{\dot{\wedge}_{\beta}}\sum_{k=1}^{\infty}2^{-k} \frac {1}{ \vert 2^{k}Q^{*} \vert } \int_{2^{k+1}Q^{*} } \bigl\vert f_{2}(y_{2}) \bigr\vert \,dy_{2}M_{1,\beta}(f_{1}) (x) \\ &\leq C\|b\|_{\dot{\wedge}_{\beta}}M_{1,\beta}(f_{1}) (x) M(f_{2}) (x) . \end{aligned}$$

Similarly, we deduce that

$$\begin{aligned} P_{23}&= \biggl(\frac{1}{ \vert Q \vert } \int_{Q} \bigl\vert T \bigl((b-\lambda)f_{1}^{\infty}, f_{2}^{0}\bigr) (z) \bigr\vert ^{\delta}\,dz \biggr)^{\frac{1}{\delta}} \\ &\leq\frac{C}{ \vert Q \vert } \int_{Q} \bigl\vert T \bigl((b-\lambda)f_{1}^{\infty}, f_{2}^{0}\bigr) (z) \bigr\vert \,dz \\ &\leq\frac{C}{ \vert Q \vert } \int_{Q} \int_{Q^{*}} \int_{(Q^{*})^{c}}\biggl( \int _{0}^{\infty}\bigl\vert K_{t}(z,y_{1},y_{2}) \bigr\vert ^{2}\frac{dt}{t}\biggr)^{1/ 2} \bigl\vert \bigl(b(y_{1})-\lambda\bigr)f_{1}(y_{1}) \bigr\vert \bigl\vert f_{2}(y_{2}) \bigr\vert \,dy_{2} \,dy_{1} \,dz \\ &\leq\frac{C}{ \vert Q \vert } \int_{Q} \int_{Q^{*}} \int_{(Q^{*})^{c}}\frac {1}{( \vert z-y_{1} \vert + \vert z-y_{2} \vert )^{2n}} \bigl\vert \bigl(b(y_{1})- \lambda\bigr)f_{1}(y_{1}) \bigr\vert \bigl\vert f_{2}(y_{2}) \bigr\vert \,dy_{2} \,dy_{1} \,dz \\ &\leq C \int_{(Q^{*})^{c}}\frac{ \vert (b(y_{1})-\lambda )f_{1}(y_{1}) \vert \,dy_{1}}{ \vert y_{1}-x_{Q} \vert ^{2n}} \int_{ Q^{*}} \bigl\vert f_{2}(y_{2}) \bigr\vert \,dy_{2} \\ &\leq C \bigl\vert Q^{*} \bigr\vert \sum_{k=1}^{\infty}\int_{2^{k+1}Q^{*}\setminus 2^{k}Q^{*}}\frac{ \vert (b(y_{1})-\lambda )f_{1}(y_{1}) \vert \,dy_{1}}{ \vert y_{1}-x_{Q} \vert ^{2n}}M(f_{2}) (x) \\ &\leq C\|b\|_{\dot{\wedge}_{\beta}} \bigl\vert Q^{*} \bigr\vert \sum _{k=1}^{\infty}\int _{2^{k+1}Q^{*}\setminus2^{k}Q^{*}} \vert y_{1}-x_{Q} \vert ^{\beta -2n} \bigl\vert f_{1}(y_{1}) \bigr\vert \,dy_{1}M(f_{2}) (x) \\ &\leq C\|b\|_{\dot{\wedge}_{\beta}} \bigl\vert Q^{*} \bigr\vert \sum _{k=1}^{\infty}\bigl\vert 2^{k}Q^{*} \bigr\vert ^{\beta / n-2} \int_{2^{k+1}Q^{*}\setminus2^{k}Q^{*}} \bigl\vert f_{1}(y_{1}) \bigr\vert \,dy_{1}M(f_{2}) (x) \\ &\leq C\|b\|_{\dot{\wedge}_{\beta}}\sum_{k=1}^{\infty}2^{-k} \frac {1}{ \vert 2^{k}Q^{*} \vert ^{1-\beta/ n}} \int_{2^{k+1}Q^{*} } \bigl\vert f_{1}(y_{1}) \bigr\vert \,dy_{1}M(f_{2}) (x) \\ &\leq C\|b\|_{\dot{\wedge}_{\beta}}M_{1,\beta}(f_{1}) (x) M(f_{2}) (x) . \end{aligned}$$

Since $(\mathbb{R}^{n} \setminus Q^{*})^{2}\subseteq\mathbb {R}^{2n}\setminus(Q^{*})^{2}\subseteq\bigcup_{k=1}^{\infty}(2^{k+3 }\sqrt {n}Q)^{2}\setminus(2^{k+2 }\sqrt{n}Q)^{2}$, we can use Minkowski’s inequality and (1.2) and (3.3) to get

$$\begin{aligned} P_{24}&\leq\frac{C}{ \vert Q \vert } \int_{Q} \bigl\vert T\bigl((b-\lambda)f_{1}^{\infty}, f_{2}^{\infty}\bigr) (z)- T\bigl((b-\lambda)f_{1}^{\infty}, f_{2}^{\infty}\bigr) (x) \bigr\vert \,dz \\ &\leq\frac{C}{ \vert Q \vert } \int_{Q} \int_{(\mathbb{R}^{n}\setminus Q^{*})^{2}} \biggl( \int_{0}^{\infty}\bigl\vert K_{t}(z, \vec{y})-K_{t}(x,\vec{y}) \bigr\vert ^{2} \frac {dt}{t}\biggr)^{1/ 2} \\ &\quad {}\times\Biggl\vert \bigl(b(y_{1})- \lambda\bigr)\prod_{i=1}^{2} f_{i}^{\infty}(y_{i}) \Biggr\vert \,d\vec{y} \,dz \\ &\leq\frac{C}{ \vert Q \vert } \int_{Q} \int_{(\mathbb{R}^{n}\setminus Q^{*})^{2}}\frac {1}{( \vert x-y_{1} \vert + \vert x-y_{2} \vert )^{2n}}\omega\biggl(\frac { \vert z-x_{Q} \vert }{ \vert x-y_{1} \vert + \vert x-y_{2} \vert } \biggr) \\ &\quad {}\times\Biggl\vert \bigl(b(y_{1})-\lambda\bigr)\prod _{i=1}^{2} f_{i}^{\infty}(y_{i}) \Biggr\vert \,d\vec{y}\,dz \\ &\leq\frac{C}{ \vert Q \vert } \int_{Q}\sum_{k=1}^{\infty}\int_{(2^{k+3 }\sqrt {n}Q\setminus2^{k+2}\sqrt{n}Q)^{2}}\frac{1}{( \vert 2^{k+3 }\sqrt {n}Q \vert )^{2}}\omega\bigl(2^{-k}\bigr) \\ &\quad {}\times \Biggl\vert \bigl(b(y_{1})-\lambda\bigr)\prod _{i=1}^{2} f_{i}^{\infty}(y_{i}) \Biggr\vert \,d\vec{y}\,dz \\ &\leq C\frac{\|Q\|_{\dot{\wedge}_{\beta}}}{ \vert Q \vert } \int_{Q}\sum_{k=1}^{\infty}\frac{1}{( \vert 2^{k+3 }\sqrt{n}Q \vert )^{2}} \int_{(2^{k+3 }\sqrt{n}Q\setminus2^{k+2}\sqrt{n}Q)^{2}}\omega \bigl(2^{-k}\bigr) \vert y_{1}-x_{Q} \vert ^{\beta} \\ &\quad {}\times\prod _{i=1}^{2} \bigl\vert f_{i}^{\infty}(y_{i}) \bigr\vert \,d\vec{y}\,dz \\ &\leq C\|b\|_{\dot{\wedge}_{\beta}} \sum_{k=1}^{\infty}\frac{\omega (2^{-k})}{( \vert 2^{k+3 }\sqrt{n}Q \vert )^{1-\beta/ n}} \int_{2^{k+3 }\sqrt {n}Q} \bigl\vert f_{1}^{\infty}(y_{1}) \bigr\vert \,dy_{1}\frac{1}{ \vert 2^{k}Q^{*} \vert } \int_{2^{k+3 }\sqrt {n}Q} \bigl\vert f_{2}^{\infty}(y_{2}) \bigr\vert \,dy_{2} \\ &\leq C\|b\|_{\dot{\wedge}_{\beta}}M_{1,\beta_{1}}(f_{1}) (x) M(f_{2}) (x). \end{aligned}$$

Thus we finish the proof of (3.2). Then Lemma 3.1 is proved. □

### Proofs of Theorem 1.1

By using Lemma 3.1 and modifying the proof of Theorem 1.1 in [8] we can finish the proof of Theorem 1.1. We omit the proof. □

## Proof of Theorems 1.2 and 1.3

For simplicity, we just consider the case $m=2$; our method still holds for general *m* with little modifications.

### Proof of Theorem 1.2

The theorem will be proved if we show that

4.1 $$ \sup_{Q}\frac{1}{|Q|^{1+\beta/ n-1/ p}} \int_{Q} \bigl\vert T_{\Pi\vec {b}}(\vec{f}) (z)- \bigl(T_{\Pi\vec {b}}(\vec{f})\bigr)_{Q} \bigr\vert \,dz\leq C \|b_{1}\|_{\dot{\wedge}_{\beta _{1}}}\|b_{2}\|_{\dot{\wedge}_{\beta _{2}}} \|f_{1}\|_{L^{p_{1}}}\|f_{2}\|_{L^{p_{2}}}. $$

Let $c=c_{1}+c_{2}+c_{3}$, which will be determined later. Then we have

$$\begin{aligned} &\frac{1}{ \vert Q \vert ^{1+\beta/ n-1/ p}} \int_{Q} \bigl\vert T_{\Pi\vec {b}}(\vec{f}) (z)- \bigl(T_{\Pi\vec {b}}(\vec{f})\bigr)_{Q} \bigr\vert \,dz \\ &\quad \leq\frac{1}{ \vert Q \vert ^{1+\beta/ n-1/ p}} \int_{Q} \bigl\vert T_{\Pi\vec {b}}(f_{1},f_{2}) (z)-c \bigr\vert \,dz \\ &\quad \leq\frac{C}{ \vert Q \vert ^{1+\beta/ n-1/ p}} \int_{Q} \bigl\vert T_{\Pi\vec {b}}\bigl(f_{1}^{0},f_{2}^{0} \bigr) (z) \bigr\vert \,dz \\ &\qquad {}+ \frac{ C}{ \vert Q \vert ^{1+\beta/ n-1/ p}} \int_{Q} \bigl\vert T_{\Pi\vec {b}}\bigl(f_{1}^{0},f_{2}^{\infty}\bigr) (z)-c_{1} \bigr\vert \,dz \\ &\qquad {}+ \frac{ C}{ \vert Q \vert ^{1+\beta/ n-1/ p}} \int_{Q} \bigl\vert T_{\Pi\vec {b}}\bigl(f_{1}^{\infty},f_{2}^{0} \bigr) (z)-c_{2} \bigr\vert \,dz \\ &\qquad {}+ \frac{ C}{ \vert Q \vert ^{1+\beta/ n-1/ p}} \int_{Q} \bigl\vert T_{\Pi\vec {b}}\bigl(f_{1}^{\infty},f_{2}^{\infty}\bigr) (z)-c_{3} \bigr\vert \,dz \\ &\quad \doteq M_{1}+M_{2}+M_{3}+M_{4}. \end{aligned}$$

We can choose $1< q, q_{j}<\infty$, $q_{j}< n/\beta_{j} < p_{j}$, $j=1,2$, with $1/q=1/q_{1}+1/q_{2}-(\beta_{1}+\beta_{2})/n$. By Hölder’s inequality and Theorem 1.1 we obtain

$$\begin{aligned} M_{1}&\leq\frac{C}{|Q|^{1+\beta/ n-1/ p}} \biggl( \int_{Q} \bigl\vert T_{\Pi\vec {b}}\bigl(f_{1}^{0},f_{2}^{0} \bigr) (z) \bigr\vert ^{q}\,dz \biggr)^{1/ q}|Q|^{1-1/ q} \\ &\leq\frac{C}{|Q|^{1+\beta/ n-1/ p}}|Q|^{1-1/ q}\bigl\| f_{1}^{0} \bigr\| _{L^{q_{1}}}\bigl\| f_{2}^{0}\bigr\| _{L^{q_{2}}} \\ &\leq C\|f_{1}\|_{L^{p_{1}}}\|f_{2}\|_{L^{p_{2}}}. \end{aligned}$$

For the second term, we take $c_{1}=T((b_{1}-\lambda_{1})f_{1}^{0},f_{2}^{\infty})(x_{Q})$. Then

$$\begin{aligned} M_{2}&\leq\frac{C}{ \vert Q \vert ^{1+\beta/ n-1/ p}} \int_{Q} \biggl( \int_{0}^{\infty}\biggl\vert \int_{Q^{*}} \int_{\mathbb{R}^{n}\setminus Q^{*}}\bigl(b_{1}(z)-\lambda _{1}\bigr) \bigl(b_{2}(z)-\lambda_{2}\bigr) \\ &\quad {}\times K_{t}(z,y_{1},y_{2})f_{1}(y_{1})f_{2}(y_{2}) \,dy_{1}\,dy_{2} \biggr\vert ^{2} \frac {dt}{t}\biggr)^{1/2}\,dz \\ &\quad {}+ \frac{C}{ \vert Q \vert ^{1+\beta/ n-1/ p}} \int_{Q} \biggl( \int_{0}^{\infty}\biggl\vert \int_{Q^{*}} \int_{\mathbb{R}^{n}\setminus Q^{*}}\bigl(b_{1}(z)-\lambda _{1}\bigr) \bigl(b_{2}(y_{2})-\lambda_{2}\bigr) \\ &\quad {}\times K_{t}(z,y_{1},y_{2})f_{1}(y_{1})f_{2}(y_{2}) \,dy_{1}\,dy_{2} \biggr\vert ^{2} \frac {dt}{t}\biggr)^{1/2}\,dz \\ &\quad {}+ \frac{C}{ \vert Q \vert ^{1+\beta/ n-1/ p}} \int_{Q} \biggl( \int_{0}^{\infty}\biggl\vert \int_{Q^{*}} \int_{\mathbb{R}^{n}\setminus Q^{*}}\bigl(b_{1}(y_{1})-\lambda _{1}\bigr) \bigl(b_{2}(z)-\lambda_{2}\bigr) \\ &\quad {}\times K_{t}(z,y_{1},y_{2})f_{1}(y_{1})f_{2}(y_{2}) \,dy_{1}\,dy_{2} \biggr\vert ^{2} \frac {dt}{t}\biggr)^{1/2}\,dz \\ &\quad {}+ \frac{C}{ \vert Q \vert ^{1+\beta/ n-1/ p}} \int_{Q} \biggl( \int_{0}^{\infty}\biggl\vert \int_{Q^{*}} \int_{\mathbb{R}^{n}\setminus Q^{*}}\bigl(b_{1}(y_{1})-\lambda _{1}\bigr) \bigl(b_{2}(y_{2})-\lambda_{2} \bigr) \\ &\quad {}\times \bigl[K_{t}(z,y_{1},y_{2})-K_{t}(x_{Q},y_{1},y_{2}) \bigr]f_{1}(y_{1})f_{2}(y_{2}) \,dy_{1}\,dy_{2} \biggr\vert ^{2}\frac{dt}{t} \biggr)^{1/2}\,dz \\ &\doteq M_{21}+M_{22}+M_{23}+M_{24}. \end{aligned}$$

By Minkowski’s inequality and the size condition (1.1) we have

$$\begin{aligned} M_{21}&\leq\frac{C}{ \vert Q \vert ^{1+\beta/ n-1/ p}} \int_{Q} \int_{Q^{*}} \int _{\mathbb{R}^{n}\setminus Q^{*}} \bigl\vert \bigl(b_{1}(z)- \lambda_{1}\bigr) \bigl(b_{2}(z)-\lambda_{2}\bigr) \bigr\vert \\ &\quad {}\times \biggl( \int_{0}^{\infty}\bigl\vert K_{t}(z,y_{1},y_{2}) \bigr\vert ^{2}\frac {dt}{t}\biggr)^{1/2} \bigl\vert f_{1}(y_{1})f_{2}(y_{2}) \bigr\vert \,dy_{1}\,dy_{2}\,dz \\ &\leq\frac{C}{ \vert Q \vert ^{1+\beta/ n-1/ p}} \int_{Q} \int_{Q^{*}} \int _{\mathbb{R}^{n}\setminus Q^{*}} \bigl\vert \bigl(b_{1}(z)- \lambda_{1}\bigr) \bigl(b_{2}(z)-\lambda_{2}\bigr) \bigr\vert \\ &\quad {}\times \frac{1}{( \vert z-y_{1} \vert + \vert z-y_{2} \vert )^{2n}} \bigl\vert f_{1}(y_{1})f_{2}(y_{2}) \bigr\vert \,dy_{1}\,dy_{2}\,dz \\ &\leq\frac{C}{ \vert Q \vert ^{1+\beta/ n-1/ p}} \int_{Q} \bigl\vert \bigl(b_{1}(z)-\lambda _{1}\bigr) \bigl(b_{2}(z)-\lambda_{2}\bigr) \bigr\vert \\ &\quad {}\times \int_{Q^{*}} \int_{\mathbb {R}^{n}\setminus Q^{*}} \frac {1}{( \vert z-y_{1} \vert + \vert z-y_{2} \vert )^{2n}} \bigl\vert f_{1}(y_{1})f_{2}(y_{2}) \bigr\vert \,dy_{1}\,dy_{2}\,dz \\ &\leq\|b_{1}\|_{\dot{\wedge}_{\beta_{1}}}\|b_{2}\|_{\dot{\wedge}_{\beta _{2}}} \vert Q \vert ^{1/ p} \int_{Q^{*}} \bigl\vert f_{1}(y_{1}) \bigr\vert \,dy_{1} \\ &\quad {}\times\sum_{k=1}^{\infty}\int _{2^{k+3}\sqrt{n}Q \setminus2^{k+2}\sqrt{n}Q } \frac { \vert f_{2}(y_{2}) \vert }{ \vert y_{2}-x_{Q} \vert ^{2n}}\,dy_{2} \\ &\leq\|b_{1}\|_{\dot{\wedge}_{\beta_{1}}}\|b_{2}\|_{\dot{\wedge}_{\beta _{2}}} |Q|^{1/ p} \int_{Q^{*}} \bigl\vert f_{1}(y_{1}) \bigr\vert \,dy_{1} \\ &\quad {}\times\sum_{k=1}^{\infty}\frac {1}{ \vert 2^{k+3}\sqrt{n}Q \vert ^{2}} \int_{2^{k+3}\sqrt{n}Q \setminus 2^{k+2}\sqrt{n}Q } \bigl\vert f_{2}(y_{2}) \bigr\vert \,dy_{2} \\ &\leq\|b_{1}\|_{\dot{\wedge}_{\beta_{1}}}\|b_{2}\|_{\dot{\wedge}_{\beta _{2}}} \|f_{1}\|_{L^{p_{1}}}\|f_{2}\|_{L^{p_{2}}}\sum _{k=1}^{\infty}2^{kn(-1-1/p_{2})} \\ &\leq\|b_{1}\|_{\dot{\wedge}_{\beta_{1}}}\|b_{2}\|_{\dot{\wedge}_{\beta _{2}}} \|f_{1}\|_{L^{p_{1}}}\|f_{2}\|_{L^{p_{2}}}. \end{aligned}$$

We now proceed as in the estimate of $M_{21}$:

$$\begin{aligned} M_{22}&\leq\frac{C}{ \vert Q \vert ^{1+\beta/ n-1/ p}} \int_{Q} \int_{Q^{*}} \int _{\mathbb{R}^{n}\setminus Q^{*}} \bigl\vert \bigl(b_{1}(z)- \lambda_{1}\bigr) \bigl(b_{2}(y_{2})-\lambda _{2}\bigr) \bigr\vert \\ & \quad {}\times\biggl( \int_{0}^{\infty}\bigl\vert K_{t}(z,y_{1},y_{2}) \bigr\vert ^{2}\frac {dt}{t}\biggr)^{1/2} \bigl\vert f_{1}(y_{1})f_{2}(y_{2}) \bigr\vert \,dy_{1}\,dy_{2}\,dz \\ &\leq\frac{C}{ \vert Q \vert ^{1+\beta/ n-1/ p}} \int_{Q} \int_{Q^{*}} \int _{\mathbb{R}^{n}\setminus Q^{*}} \bigl\vert \bigl(b_{1}(z)- \lambda_{1}\bigr) \bigl(b_{2}(y_{2})-\lambda _{2}\bigr) \bigr\vert \\ &\quad {}\times \frac{1}{( \vert z-y_{1} \vert + \vert z-y_{2} \vert )^{2n}} \bigl\vert f_{1}(y_{1})f_{2}(y_{2}) \bigr\vert \,dy_{1}\,dy_{2}\,dz \\ &\leq\frac{C}{ \vert Q \vert ^{1+\beta/ n-1/ p}} \int_{Q} \bigl\vert \bigl(b_{1}(z)- \lambda_{1}\bigr) \bigr\vert \\ & \quad {}\times \int_{Q^{*}} \int_{\mathbb{R}^{n}\setminus Q^{*}} \frac { \vert (b_{2}(z)-\lambda _{2}) \vert }{( \vert z-y_{1} \vert + \vert z-y_{2} \vert )^{2n}} \bigl\vert f_{1}(y_{1})f_{2}(y_{2}) \bigr\vert \,dy_{1}\,dy_{2}\,dz \\ &\leq\frac{\|b_{1}\|_{\dot{\wedge}_{\beta_{1}}}\|b_{2}\|_{\dot{\wedge }_{\beta_{2}}}}{ \vert Q \vert ^{\beta_{2}/n-1/ p}} \int_{Q^{*}} \bigl\vert f_{1}(y_{1}) \bigr\vert \,dy_{1}\sum_{k=1}^{\infty}\int_{2^{k+3}\sqrt{n}Q \setminus2^{k+2}\sqrt{n}Q } \frac{ \vert f_{2}(y_{2}) \vert }{ \vert y_{2}-x_{Q} \vert ^{2n-\beta_{2}}}\,dy_{2} \\ &\leq\|b_{1}\|_{\dot{\wedge}_{\beta_{1}}}\|b_{2}\|_{\dot{\wedge}_{\beta _{2}}} \|f_{1}\|_{L^{p_{1}}}\|f_{2}\|_{L^{p_{2}}}\sum _{k=1}^{\infty}2^{kn(-1-1/p_{2}+\beta_{2}/n)} \\ &\leq\|b_{1}\|_{\dot{\wedge}_{\beta_{1}}}\|b_{2}\|_{\dot{\wedge}_{\beta _{2}}} \|f_{1}\|_{L^{p_{1}}}\|f_{2}\|_{L^{p_{2}}} \end{aligned}$$

because of $-1-1/p_{2} +\beta_{2}/n<0$.

Similarly,

$$ M_{23}\leq C\|b_{1}\|_{\dot{\wedge}_{\beta_{1}}}\|b_{2} \|_{\dot{\wedge }_{\beta_{2}}} \|f_{1}\|_{L^{p_{1}}}\|f_{2} \|_{L^{p_{2}}}. $$

By Minkowski’s inequality and (1.2) we have

$$\begin{aligned} M_{24}&\leq\frac{C}{ \vert Q \vert ^{1+\beta/ n-1/ p}} \int_{Q} \int_{Q^{*}} \int _{\mathbb{R}^{n}\setminus Q^{*}} \bigl\vert \bigl(b_{1}(z)- \lambda_{1}\bigr) \bigl(b_{2}(y_{2})-\lambda _{2}\bigr) \bigr\vert \\ &\quad {}\times \biggl( \int_{0}^{\infty}\bigl\vert K_{t}(z,y_{1},y_{2})-K_{t}(x_{Q},y_{1},y_{2}) \bigr\vert ^{2}\frac{dt}{t} \biggr)^{1/2} \bigl\vert f_{1}(y_{1})f_{2}(y_{2}) \bigr\vert \,dy_{1}\,dy_{2}\,dz \\ &\leq C\|b_{1}\|_{\dot{\wedge}_{\beta_{1}}}\|b_{2}\|_{\dot{\wedge }_{\beta_{2}}} \frac{1}{ \vert Q \vert ^{1+\beta/ n-1/ p}} \int_{Q} \int_{Q^{*}} \int _{\mathbb{R}^{n}\setminus Q^{*}} \bigl\vert \bigl(b_{1}(z)- \lambda_{1}\bigr) \bigl(b_{2}(y_{2})-\lambda _{2}\bigr) \bigr\vert \\ &\quad {}\times \frac{\omega(\frac { \vert z-x_{Q} \vert }{ \vert z-y_{1} \vert + \vert z-y_{2} \vert })}{( \vert z-y_{1} \vert + \vert z-y_{2} \vert )^{2n}} \bigl\vert f_{1}(y_{1})f_{2}(y_{2}) \bigr\vert \,dy_{1}\,dy_{2}\,dz \\ &\leq C\|b_{1}\|_{\dot{\wedge}_{\beta_{1}}}\|b_{2}\|_{\dot{\wedge }_{\beta_{2}}} \frac{1}{ \vert Q \vert ^{1+\beta_{2}/ n-1/ p}} \int_{Q} \int_{Q^{*}} \int _{\mathbb{R}^{n}\setminus Q^{*}} \bigl\vert \bigl(b_{1}(z)- \lambda_{1}\bigr) \bigl(b_{2}(y_{2})-\lambda _{2}\bigr) \bigr\vert \\ &\quad {}\times \frac{\omega(\frac{ \vert z-x_{Q} \vert }{ \vert x_{Q}-y_{2} \vert })}{( \vert z-y_{1} \vert + \vert z-y_{2} \vert )^{2n-\beta _{2}}} \bigl\vert f_{1}(y_{1})f_{2}(y_{2}) \bigr\vert \,dy_{1}\,dy_{2}\,dz \\ &\leq C\|b_{1}\|_{\dot{\wedge}_{\beta_{1}}}\|b_{2}\|_{\dot{\wedge }_{\beta_{2}}} \frac{C}{ \vert Q \vert ^{\beta_{2}/ n-1/ p}} \int_{Q} \bigl\vert \bigl(b_{1}(z)-\lambda _{1}\bigr) \bigr\vert \\ &\quad {}\times \int_{Q^{*}} \int_{\mathbb{R}^{n}\setminus Q^{*}} \frac{ \vert (b_{2}(z)-\lambda _{2}) \vert }{( \vert z-y_{1} \vert + \vert z-y_{2} \vert )^{2n}} \bigl\vert f_{1}(y_{1})f_{2}(y_{2}) \bigr\vert \,dy_{1}\,dy_{2}\,dz \\ &\leq C\frac{\|b_{1}\|_{\dot{\wedge}_{\beta_{1}}}\|b_{2}\|_{\dot{\wedge }_{\beta_{2}}}}{|Q|^{\beta_{2}/n-1/ p}} \int_{Q^{*}} \bigl\vert f_{1}(y_{1}) \bigr\vert \,dy_{1} \\ &\quad {}\times\sum_{k=1}^{\infty}\frac{\omega(2^{-k})}{|2^{k+3}\sqrt{n}Q |^{2-\beta_{2} /n}} \int_{2^{k+3}\sqrt{n}Q \setminus2^{k+2}\sqrt{n}Q } \bigl\vert f_{2}(y_{2}) \bigr\vert \,dy_{2} \\ &\leq C\|b_{1}\|_{\dot{\wedge}_{\beta_{1}}}\|b_{2}\|_{\dot{\wedge }_{\beta_{2}}} \|f_{1}\|_{L^{p_{1}}}\|f_{2}\|_{L^{p_{2}}}\sum _{k=1}^{\infty}\omega\bigl(2^{-k} \bigr)2^{kn(1-\beta_{2}/n+1/p_{2})} \\ &\leq C\|b_{1}\|_{\dot{\wedge}_{\beta_{1}}}\|b_{2}\|_{\dot{\wedge }_{\beta_{2}}} \|f_{1}\|_{L^{p_{1}}}\|f_{2}\|_{L^{p_{2}}}, \end{aligned}$$

where we have used the fact $1-\beta_{2}/n +1/p_{2}>0$.

Thus

$$ M_{2} \leq C\|b_{1}\|_{\dot{\wedge}_{\beta_{1}}}\|b_{2} \|_{\dot{\wedge }_{\beta_{2}}} \|f_{1}\|_{L^{p_{1}}}\|f_{2} \|_{L^{p_{2}}}. $$

Similarly,

$$ M_{3} \leq C\|b_{1}\|_{\dot{\wedge}_{\beta_{1}}}\|b_{2} \|_{\dot{\wedge }_{\beta_{2}}} \|f_{1}\|_{L^{p_{1}}}\|f_{2} \|_{L^{p_{2}}}. $$

We deal with $M_{4}$ as follows:

$$\begin{aligned} M_{4}&\leq\frac{C}{ \vert Q \vert ^{1+\beta/ n-1/ p}} \int_{Q} \biggl( \int_{0}^{\infty}\biggl\vert \int_{(\mathbb{R}^{n}\setminus Q^{*})^{2}}\bigl(b_{1}(z)-\lambda _{1}\bigr) \bigl(b_{2}(z)-\lambda_{2}\bigr) \\ &\quad {}\times K_{t}(z,y_{1},y_{2})f_{1}(y_{1})f_{2}(y_{2}) \,dy_{1}\,dy_{2} \biggr\vert ^{2} \frac {dt}{t}\biggr)^{1/2}\,dz \\ &\quad {}+ \frac{C}{ \vert Q \vert ^{1+\beta/ n-1/ p}} \int_{Q} \biggl( \int_{0}^{\infty}\biggl\vert \int_{(\mathbb{R}^{n}\setminus Q^{*})^{2}}\bigl(b_{1}(z)-\lambda _{1}\bigr) \bigl(b_{2}(y_{2})-\lambda_{2}\bigr) \\ &\quad {}\times \bigl[K_{t}(z,y_{1},y_{2})-K_{t}(x_{Q},y_{1},y_{2}) \bigr]f_{1}(y_{1})f_{2}(y_{2}) \,dy_{1}\,dy_{2} \biggr\vert ^{2}\frac{dt}{t} \biggr)^{1/2}\,dz \\ &\quad {}+ \frac{C}{ \vert Q \vert ^{1+\beta/ n-1/ p}} \int_{Q} \biggl( \int_{0}^{\infty}\biggl\vert \int_{(\mathbb{R}^{n}\setminus Q^{*})^{2}}\bigl(b_{1}(y_{1})-\lambda _{1}\bigr) \bigl(b_{2}(z)-\lambda_{2}\bigr) \\ &\quad {}\times \bigl[K_{t}(z,y_{1},y_{2})-K_{t}(x_{Q},y_{1},y_{2}) \bigr]f_{1}(y_{1})f_{2}(y_{2}) \,dy_{1}\,dy_{2} \biggr\vert ^{2}\frac{dt}{t} \biggr)^{1/2}\,dz \\ &\quad {}+ \frac{C}{ \vert Q \vert ^{1+\beta/ n-1/ p}} \int_{Q} \biggl( \int_{0}^{\infty}\biggl\vert \int_{(\mathbb{R}^{n}\setminus Q^{*})^{2}}\bigl(b_{1}(y_{1})-\lambda _{1}\bigr) \bigl(b_{2}(y_{2})-\lambda_{2} \bigr) \\ &\quad {}\times \bigl[K_{t}(z,y_{1},y_{2})-K_{t}(x_{Q},y_{1},y_{2}) \bigr]f_{1}(y_{1})f_{2}(y_{2}) \,dy_{1}\,dy_{2} \biggr\vert ^{2}\frac{dt}{t} \biggr)^{1/2}\,dz \\ &\doteq M_{41}+M_{42}+M_{43}+M_{44}. \end{aligned}$$

By Minkowski’s inequality and the size condition (1.1) we have

$$\begin{aligned} M_{41}&\leq\frac{C}{ \vert Q \vert ^{1+\beta/ n-1/ p}} \int_{Q} \int_{(\mathbb {R}^{n}\setminus Q^{*})^{2}} \bigl\vert \bigl(b_{1}(z)- \lambda_{1}\bigr) \bigl(b_{2}(z)-\lambda_{2}\bigr) \bigr\vert \\ &\quad {}\times \biggl( \int_{0}^{\infty}\bigl\vert K_{t}(z,y_{1},y_{2}) \bigr\vert ^{2}\frac {dt}{t}\biggr)^{1/2} \bigl\vert f_{1}(y_{1})f_{2}(y_{2}) \bigr\vert \,dy_{1}\,dy_{2}\,dz \\ &\leq\frac{C}{ \vert Q \vert ^{1+\beta/ n-1/ p}} \int_{Q} \int_{(\mathbb {R}^{n}\setminus Q^{*})^{2}} \bigl\vert \bigl(b_{1}(z)- \lambda_{1}\bigr) \bigl(b_{2}(z)-\lambda_{2}\bigr) \bigr\vert \\ &\quad {}\times \frac{1}{( \vert z-y_{1} \vert + \vert z-y_{2} \vert )^{2n}} \bigl\vert f_{1}(y_{1})f_{2}(y_{2}) \bigr\vert \,dy_{1}\,dy_{2}\,dz \\ &\leq\|b_{1}\|_{\dot{\wedge}_{\beta_{1}}}\|b_{2}\|_{\dot{\wedge}_{\beta _{2}}} \vert Q \vert ^{1/ p} \sum_{k=1}^{\infty}\int_{2^{k+3}\sqrt{n}Q \setminus 2^{k+2}\sqrt{n}Q } \frac{ \vert f_{1}(y_{1}) \vert }{ \vert y_{1}-x_{Q} \vert ^{n}}\,dy_{1} \\ &\quad {}\times\sum _{k=1}^{\infty}\int_{2^{k+3}\sqrt{n}Q \setminus2^{k+2}\sqrt{n}Q } \frac{ \vert f_{2}(y_{2}) \vert }{ \vert y_{2}-x_{Q} \vert ^{n}}\,dy_{2} \\ &\leq\|b_{1}\|_{\dot{\wedge}_{\beta_{1}}}\|b_{2}\|_{\dot{\wedge}_{\beta _{2}}} \|f_{1}\|_{L^{p_{1}}}\|f_{2}\|_{L^{p_{2}}} |Q|^{1/ p} \sum_{k=1}^{\infty}\bigl\vert 2^{k+3}\sqrt{n}Q \bigr\vert ^{-1/p_{1}}\sum _{k=1}^{\infty}\bigl\vert 2^{k+3}\sqrt{n}Q \bigr\vert ^{-1/p_{2}} \\ &\leq\|b_{1}\|_{\dot{\wedge}_{\beta_{1}}}\|b_{2}\|_{\dot{\wedge}_{\beta _{2}}} \|f_{1}\|_{L^{p_{1}}}\|f_{2}\|_{L^{p_{2}}}. \end{aligned}$$

By Minkowski’s inequality and the smooth condition (1.2) we have

$$\begin{aligned} M_{42}&\leq\frac{C}{ \vert Q \vert ^{1+\beta/ n-1/ p}} \int_{Q} \int_{(\mathbb {R}^{n}\setminus Q^{*})^{2}} \bigl\vert \bigl(b_{1}(z)- \lambda_{1}\bigr) \bigl(b_{2}(y_{2})- \lambda_{2}\bigr) \bigr\vert \\ &\quad {}\times \biggl( \int_{0}^{\infty}\bigl\vert K_{t}(z,y_{1},y_{2})-K_{t}(x_{Q},y_{1},y_{2}) \bigr\vert ^{2}\frac{dt}{t} \biggr)^{1/2} \bigl\vert f_{1}(y_{1})f_{2}(y_{2}) \bigr\vert \,dy_{1}\,dy_{2}\,dz \\ &\leq\frac{\|b_{1}\|_{\dot{\wedge}_{\beta_{1}}}\|b_{2}\|_{\dot{\wedge }_{\beta_{2}}}}{ \vert Q \vert ^{\beta_{2}/ n-1/ p}} \sum_{k=1}^{\infty}\int _{2^{k+3}\sqrt{n}Q \setminus2^{k+2}\sqrt{n}Q } \frac { \vert f_{1}(y_{1}) \vert }{ \vert y_{1}-x_{Q} \vert ^{n}}\,dy_{1} \\ &\quad {}\times\sum _{i=1}^{\infty}\int_{2^{i+3}\sqrt {n}Q \setminus2^{i+2}\sqrt{n}Q } \frac{ \vert f_{2}(y_{2}) \vert \omega (2^{-i})}{ \vert y_{2}-x_{Q} \vert ^{n-\beta_{2}}}\,dy_{2} \\ &\leq\|b_{1}\|_{\dot{\wedge}_{\beta_{1}}}\|b_{2}\|_{\dot{\wedge}_{\beta _{2}}} \|f_{1}\|_{L^{p_{1}}}\|f_{2}\|_{L^{p_{2}}} \sum _{k=1}^{\infty}\bigl\vert 2^{k+3} \sqrt {n}Q \bigr\vert ^{-1/p_{1}} \vert Q \vert ^{1/ p} \\ &\quad {}\times \sum_{i=1}^{\infty}\omega \bigl(2^{-i}\bigr) \bigl\vert 2^{i+3}\sqrt{n}Q \bigr\vert ^{\beta _{2}/n-1/p_{2}} \vert Q \vert ^{-\beta_{2}/n+1/p_{2}} \\ &\leq\|b_{1}\|_{\dot{\wedge}_{\beta_{1}}}\|b_{2}\|_{\dot{\wedge}_{\beta _{2}}} \|f_{1}\|_{L^{p_{1}}}\|f_{2}\|_{L^{p_{2}}}\sum _{i=1}^{\infty}\omega \bigl(2^{-i} \bigr)2^{in(\beta/ n-1/ p)} \\ &\leq\|b_{1}\|_{\dot{\wedge}_{\beta_{1}}}\|b_{2}\|_{\dot{\wedge}_{\beta _{2}}} \|f_{1}\|_{L^{p_{1}}}\|f_{2}\|_{L^{p_{2}}}. \end{aligned}$$

Similarly,

$$ M_{43}\leq C\|b_{1}\|_{\dot{\wedge}_{\beta_{1}}}\|b_{2} \|_{\dot{\wedge }_{\beta_{2}}} \|f_{1}\|_{L^{p_{1}}}\|f_{2} \|_{L^{p_{2}}}. $$

Now we estimate $M_{44}$:

$$\begin{aligned} M_{44}&\leq\frac{C}{ \vert Q \vert ^{1+\beta/ n-1/ p}} \int_{Q} \int_{(\mathbb {R}^{n}\setminus Q^{*})^{2}} \bigl\vert \bigl(b_{1}(y_{1})- \lambda_{1}\bigr) \bigl(b_{2}(y_{2})- \lambda_{2}\bigr) \bigr\vert \\ &\quad {}\times \biggl( \int_{0}^{\infty}\bigl\vert K_{t}(z,y_{1},y_{2})-K_{t}(x_{Q},y_{1},y_{2}) \bigr\vert ^{2}\frac{dt}{t} \biggr)^{1/2} \bigl\vert f_{1}(y_{1})f_{2}(y_{2}) \bigr\vert \,dy_{1}\,dy_{2}\,dz \\ &\leq\frac{\|b_{1}\|_{\dot{\wedge}_{\beta_{1}}}\|b_{2}\|_{\dot{\wedge }_{\beta_{2}}}}{ \vert Q \vert ^{\beta/ n-1/ p}} \sum_{k=1}^{\infty}\int _{(2^{k+3}\sqrt{n}Q)^{2} \setminus(2^{k+2}\sqrt{n}Q)^{2} } \frac { \vert f_{1}(y_{1}) \vert }{ \vert y_{2}-x_{Q} \vert ^{2n-\beta_{1}-\beta_{2}}} \\ &\quad {}\times\omega\biggl(\frac { \vert z-x_{Q} \vert }{ \vert y_{2}-x_{Q} \vert } \biggr)\,dy_{1}\,dy_{2} \\ &\leq\|b_{1}\|_{\dot{\wedge}_{\beta_{1}}}\|b_{2}\|_{\dot{\wedge}_{\beta _{2}}} \|f_{1}\|_{L^{p_{1}}}\|f_{2}\|_{L^{p_{2}}}\sum _{k=1}^{\infty}\omega \bigl(2^{-k} \bigr)2^{kn(\beta/ n-1/ p)} \\ &\leq\|b_{1}\|_{\dot{\wedge}_{\beta_{1}}}\|b_{2}\|_{\dot{\wedge}_{\beta _{2}}} \|f_{1}\|_{L^{p_{1}}}\|f_{2}\|_{L^{p_{2}}}. \end{aligned}$$

Combing the estimates for $M_{1}$, $M_{2}$, $M_{3}$, $M_{4}$, we get (4.1). Thus the proof of Theorem 1.2 is completed. □

### Proof of Theorem 1.3

Let $c=c_{1}+c_{2}+c_{3}$, which will be determined later. Then we have

$$\begin{aligned} &\frac{1}{ \vert Q \vert ^{1+\beta/ n}} \int_{Q} \bigl\vert T_{\Pi\vec {b}}(\vec{f}) (z)- \bigl(T_{\Pi\vec {b}}(\vec{f})\bigr)_{Q} \bigr\vert \,dz \\ &\quad \leq\frac{1}{ \vert Q \vert ^{1+\beta/ n}} \int_{Q} \bigl\vert T_{\Pi\vec {b}}(f_{1},f_{2}) (z)-c \bigr\vert \,dz \\ &\quad \leq\frac{C}{ \vert Q \vert ^{1+\beta/ n}} \int_{Q} \bigl\vert \bigl(b_{1}(z)-\lambda _{1}\bigr) \bigl(b_{2}(z)-\lambda_{2} \bigr)T(f_{1},f_{2}) (z) \bigr\vert \,dz \\ &\qquad {}+ \frac{ C}{ \vert Q \vert ^{1+\beta/ n}} \int_{Q} \bigl\vert \bigl(b_{2}(z)- \lambda_{2}\bigr)T_{\vec {b}}^{1}(f_{1},f_{2}) (z)-c_{1} \bigr\vert \,dz \\ &\qquad {}+ \frac{ C}{ \vert Q \vert ^{1+\beta/ n}} \int_{Q} \bigl\vert \bigl(b_{1}(z)- \lambda_{1}\bigr)T_{\vec {b}}^{2}(f_{1},f_{2}) (z)-c_{2} \bigr\vert \,dz \\ &\qquad {}+ \frac{ C}{ \vert Q \vert ^{1+\beta/ n}} \int_{Q} \bigl\vert T\bigl((b_{1}-\lambda _{1})f_{1},(b_{2}-\lambda_{2})f_{2} \bigr) (z)-c_{3} \bigr\vert \,dz \\ &\quad \doteq N_{1}+N_{2}+N_{3}+N_{4}. \end{aligned}$$

In what follows, we estimate each term separately. For $1< r< p$, by the Hölder inequality we have

$$ N_{1}\leq C\|b_{1}\|_{\dot{\wedge}_{\beta_{1}}}\|b_{2} \|_{\dot{\wedge }_{\beta_{2}}}M_{r}\bigl(T(f_{1},f_{2})\bigr) (x). $$

Observe that

$$\begin{aligned} &[b_{1},T](f_{1},f_{2}) (z) \\ &\quad < \bigl\vert \bigl(b_{1}(z)-\lambda _{1}\bigr) \bigr\vert T(f_{1},f_{2}) (z)+T\bigl(f_{1}^{0},f_{2}^{0} \bigr) (z) \\ &\qquad {}+ \biggl( \int_{0}^{\infty}\biggl\vert \int_{(\mathbb {R}^{n})^{m}}\bigl(b_{1}(y_{1})- \lambda_{1}\bigr)K_{t}(x,y_{1},y_{2}) f_{1}^{\infty}(y_{1})f_{2}^{0}(y_{2}) \,dy_{1}\,dy_{2} \biggr\vert ^{2}\frac{dt}{t} \biggr)^{\frac{1}{2}} \\ &\qquad {}+ \biggl( \int_{0}^{\infty}\biggl\vert \int_{(\mathbb {R}^{n})^{m}}\bigl(b_{1}(y_{1})- \lambda_{1}\bigr)K_{t}(x,y_{1},y_{2}) f_{1}^{0}(y_{1})f_{2}^{\infty}(y_{2})\,dy_{1}\,dy_{2} \biggr\vert ^{2}\frac{dt}{t} \biggr)^{\frac{1}{2}} \\ &\qquad {}+ \biggl( \int_{0}^{\infty}\biggl\vert \int_{(\mathbb {R}^{n})^{m}}\bigl(b_{1}(y_{1})- \lambda_{1}\bigr)K_{t}(x,y_{1},y_{2}) f^{\infty}_{1}(y_{1})f^{\infty}_{2}(y_{2})\,dy_{1}\,dy_{2} \biggr\vert ^{2}\frac{dt}{t} \biggr)^{\frac{1}{2}}. \end{aligned}$$

Let

$$\begin{aligned} c_{1}'&=\|b_{2}\|_{\dot{\wedge}_{\beta_{2}}} \vert Q \vert ^{\beta_{2}/ n} \\ &\quad {}\times\biggl( \int _{0}^{\infty}\biggl\vert \int_{(\mathbb{R}^{n})^{m}}\bigl(b_{1}(y_{1})-\lambda _{1}\bigr)K_{t}(x,y_{1},y_{2}) f_{1}^{\infty}(y_{1})f_{2}^{0}(y_{2}) \,dy_{1}\,dy_{2} \biggr\vert ^{2} \frac {dt}{t} \biggr)^{\frac{1}{2}} \\ &\quad {}+\|b_{2}\|_{\dot{\wedge}_{\beta_{2}}} \vert Q \vert ^{\beta_{2}/ n} \\ &\quad {}\times \biggl( \int _{0}^{\infty}\biggl\vert \int_{(\mathbb{R}^{n})^{m}}\bigl(b_{1}(y_{1})-\lambda _{1}\bigr)K_{t}(x,y_{1},y_{2}) f_{1}^{0}(y_{1})f_{2}^{\infty}(y_{2}) \,dy_{1}\,dy_{2} \biggr\vert ^{2} \frac {dt}{t} \biggr)^{\frac{1}{2}} \\ &\quad {}+\|b_{2}\|_{\dot{\wedge}_{\beta_{2}}} \vert Q \vert ^{\beta_{2}/ n} \\ &\quad {}\times\biggl( \int _{0}^{\infty}\biggl\vert \int_{(\mathbb{R}^{n})^{m}}\bigl(b_{1}(y_{1})-\lambda _{1}\bigr)K_{t}(x,y_{1},y_{2}) f^{\infty}_{1}(y_{1})f^{\infty}_{2}(y_{2}) \,dy_{1}\,dy_{2} \biggr\vert ^{2}\frac{dt}{t} \biggr)^{\frac{1}{2}}. \end{aligned}$$

Then

$$\begin{aligned} N_{2}&\leq\frac{C}{ \vert Q|^{1+\beta/ n}} \int_{Q} \bigl\vert \|b_{2}\|_{\dot {\wedge}_{\beta_{2}}}|Q|^{\beta_{2}/ n} [b_{1},T](f_{1},f_{2}) (z)-c_{1}' \bigr\vert \,dz \\ &\leq\frac{C\|b_{2}\|_{\dot{\wedge}_{\beta_{2}}}}{ \vert Q \vert ^{1+\beta_{1}/ n}} \int_{Q} \bigl\vert \bigl(b_{1}(z)- \lambda_{1}\bigr) \bigr\vert T(f_{1},f_{2}) (z) \,dz \\ &\quad {}+ \frac{C\|b_{2}\|_{\dot{\wedge}_{\beta_{2}}}}{ \vert Q \vert ^{1+\beta_{1}/ n}} \int_{Q} T\bigl(f_{1}^{0},f_{2}^{0} \bigr) (z)\,dz \\ &\quad {}+ \frac{C\|b_{2}\|_{\dot{\wedge}_{\beta_{2}}}}{ \vert Q \vert ^{1+\beta_{1}/ n}} \int_{Q} \biggl( \int_{0}^{\infty}\biggl\vert \int_{(\mathbb {R}^{n})^{m}}\bigl(b_{1}(y_{1})- \lambda_{1}\bigr) \\ &\quad {}\times \bigl[K_{t}(z,y_{1},y_{2})- K_{t}(x_{Q},y_{1},y_{2})\bigr] f_{1}^{0}(y_{1})f_{2}^{\infty}(y_{2})\,dy_{1}\,dy_{2} \biggr\vert ^{2}\frac{dt}{t} \biggr)^{\frac{1}{2}}\,dz \\ &\quad {}+ \frac{C\|b_{2}\|_{\dot{\wedge}_{\beta_{2}}}}{|Q|^{1+\beta_{1}/ n}} \int_{Q} \biggl( \int_{0}^{\infty}\biggl\vert \int_{(\mathbb {R}^{n})^{m}}\bigl(b_{1}(y_{1})- \lambda_{1}\bigr) \\ &\quad {}\times \bigl[K_{t}(z,y_{1},y_{2})- K_{t}(x_{Q},y_{1},y_{2})\bigr] f_{1}^{\infty}(y_{1})f_{2}^{0}(y_{2}) \,dy_{1}\,dy_{2} \biggr\vert ^{2}\frac{dt}{t} \biggr)^{\frac{1}{2}}\,dz \\ &\quad {}+ \frac{C\|b_{2}\|_{\dot{\wedge}_{\beta_{2}}}}{|Q|^{1+\beta_{1}/ n}} \int_{Q} \biggl( \int_{0}^{\infty}\biggl\vert \int_{(\mathbb {R}^{n})^{m}}\bigl(b_{1}(y_{1})- \lambda_{1}\bigr) \\ &\quad {}\times \bigl[K_{t}(z,y_{1},y_{2})- K_{t}(x_{Q},y_{1},y_{2})\bigr] f_{1}^{\infty}(y_{1})f_{2}^{\infty}(y_{2}) \,dy_{1}\,dy_{2} \biggr\vert ^{2}\frac{dt}{t} \biggr)^{\frac{1}{2}}\,dz \\ &\doteq N_{21}+N_{22}+N_{23}+N_{24}+N_{25}. \end{aligned}$$

By the Hölder inequality we have

$$\begin{aligned} N_{21}&\leq C \|b_{2}\|_{\dot{\wedge}_{\beta_{2}}} \biggl( \frac {1}{|Q|^{r'\beta_{1}/ n+1}} \int_{Q} \bigl\vert b_{1}(z)- \lambda_{1} \bigr\vert ^{r'}\,dz \biggr)^{1/ r'} \biggl(\frac{1}{|Q|} \int_{Q} \bigl\vert T (f_{1},f_{2}) (z) \bigr\vert ^{r}\,dz \biggr)^{1/ r} \\ &\leq\|b_{1}\|_{\dot{\wedge}_{\beta_{1}}}\|b_{2}\|_{\dot{\wedge}_{\beta _{2}}}M_{r} \bigl( T (f_{1},f_{2})\bigr) (x). \end{aligned}$$

Take $1< q_{1}< p_{1}$, $1< q_{2}< p_{2}$, and $1< q<\infty$ such that $1/q=1/q_{1}+1/q_{2}$. Then by the Hölder inequality and Lemma 2.3 we have

$$\begin{aligned} N_{22}&\leq\frac{C \Vert b_{2} \Vert _{\dot{\wedge}_{\beta_{2}}}}{|Q|^{\beta _{1}/ n+1/ q}} \biggl( \int_{Q} |T \bigl((b_{1}-\lambda _{1})f_{1}^{0},f_{2}^{0} \bigr) (z)|^{q}\,dz \biggr)^{1/ q} \\ &\leq\frac{C \Vert b_{2} \Vert _{\dot{\wedge}_{\beta_{2}}}}{|Q|^{\beta_{1}/ n+1/ q}} \bigl\Vert (b_{1}-\lambda_{1})f_{1}^{0} \bigr\Vert _{L^{q_{1}}} \bigl\Vert f_{2}^{0} \bigr\Vert _{L^{q_{2}}} \\ &\leq\frac{C \Vert b_{1} \Vert _{\dot{\wedge}_{\beta_{1}}} \Vert b_{2} \Vert _{\dot{\wedge }_{\beta_{2}}}}{|Q|^{ 1/ q}} \bigl\Vert f_{1}^{0} \bigr\Vert _{L^{q_{1}}} \bigl\Vert f_{2}^{0} \bigr\Vert _{L^{q_{2}}} \\ &\leq \Vert b_{1} \Vert _{\dot{\wedge}_{\beta_{1}}} \Vert b_{2} \Vert _{\dot{\wedge}_{\beta _{2}}} M_{q_{1}}(f_{1}) (x)M_{q_{2}}(f_{2}) (x). \end{aligned}$$

For $y_{2}\in(Q^{*})^{c}$, $|y_{2}-x_{Q}|\sim|y_{2}-z|$, and $|z-x_{Q}|\leq\frac {|y_{2}-z|}{2}\leq\frac{1}{2} \max{\{|z-y_{1}|,|z-y_{2}|\}}$, by Minkowski’s inequality and the smooth condition (1.2) we get

$$\begin{aligned} N_{23}&\leq\frac{C\|b_{2}\|_{\dot{\wedge}_{\beta_{2}}}}{ \vert Q \vert ^{1+\beta _{1}/ n}} \int_{Q} \int_{(\mathbb{R}^{n})^{2}} \bigl\vert \bigl(b_{1}(y_{1})- \lambda_{1}\bigr) \bigr\vert \\ &\quad {}\times \biggl( \int_{0}^{\infty}\bigl\vert K_{t}(z,y_{1},y_{2})- K_{t}(x_{Q},y_{1},y_{2}) \bigr\vert ^{2}\frac{dt}{t} \biggr)^{\frac{1}{2}} \bigl\vert f_{1}^{0}(y_{1})f_{2}^{\infty}(y_{2}) \bigr\vert \,dy_{1}\,dy_{2} \,dz \\ &\leq\frac{C\|b_{1}\|_{\dot{\wedge}_{\beta_{1}}}\|b_{2}\|_{\dot{\wedge }_{\beta_{2}}}}{ \vert Q \vert ^{1+\beta_{1}/ n}} \int_{Q} \int_{(\mathbb {R}^{n})^{2}}\frac{ \vert y_{1}-x_{Q} \vert ^{\beta_{1}}}{( \vert z-y_{1} \vert + \vert z-y_{2} \vert )^{2n}} \\ &\quad {}\times \omega\biggl(\frac{ \vert z-x_{Q} \vert }{ \vert z-y_{1} \vert + \vert z-y_{2} \vert }\biggr) \bigl\vert f_{1}^{0}(y_{1})f_{2}^{\infty}(y_{2}) \bigr\vert \,dy_{1}\,dy_{2} \,dz \\ &\leq\frac{C\|b_{1}\|_{\dot{\wedge}_{\beta_{1}}}\|b_{2}\|_{\dot{\wedge }_{\beta_{2}}}}{ \vert Q \vert ^{1+\beta_{1}/ n}} \int_{Q} \int_{(\mathbb {R}^{n})^{2}}\frac{ \vert f_{1}^{0}(y_{1})f_{2}^{\infty}(y_{2}) \vert }{( \vert z-y_{1} \vert + \vert z-y_{2} \vert )^{2n-\beta_{1}}} \\ &\quad {}\times \omega\biggl(\frac{ \vert z-x_{Q} \vert }{ \vert z-y_{1} \vert + \vert z-y_{2} \vert }\biggr)\,dy_{1} \,dy_{2} \,dz \\ &\leq\frac{C}{ \vert Q \vert }\|b_{1}\|_{\dot{\wedge}_{\beta_{1}}}\|b_{2} \|_{\dot {\wedge}_{\beta_{2}}} \int_{Q} \int_{Q^{*}} \bigl\vert f_{1}(y_{1}) \bigr\vert \int_{(Q^{*})^{c} }\frac{ \vert f_{2}(y_{2}) \vert }{ \vert z-y_{2} \vert ^{2n}} \omega\biggl(\frac{ \vert z-x_{Q} \vert }{ \vert z-y_{2} \vert } \biggr)\,dy_{2} \,dy_{1}\,dz \\ &\leq\frac{C}{ \vert Q \vert }\|b_{1}\|_{\dot{\wedge}_{\beta_{1}}}\|b_{2} \|_{\dot {\wedge}_{\beta_{2}}} \int_{Q} \int_{Q^{*}} \bigl\vert f_{1}(y_{1}) \bigr\vert \\ &\quad {}\times\sum_{k=1}^{\infty}\int_{2^{k+3}\sqrt{n} Q\setminus2^{k+2}\sqrt{n} Q} \bigl\vert f_{2}(y_{2}) \bigr\vert \bigl\vert 2^{k}\sqrt{n}Q \bigr\vert ^{-2} \omega \bigl(2^{-k}\bigr)\,dy_{2} \,dy_{1}\,dz \\ &\leq C\|b_{1}\|_{\dot{\wedge}_{\beta_{1}}}\|b_{2}\|_{\dot{\wedge }_{\beta_{2}}} \frac{1}{ \vert Q \vert } \int_{Q^{*}} \bigl\vert f_{1}(y_{1}) \bigr\vert \,dy_{1} \\ &\quad {}\times \sum_{k=1}^{\infty} \vert Q \vert \bigl\vert 2^{k+3}\sqrt{n} Q \bigr\vert ^{-1} \omega\bigl(2^{-k}\bigr) \frac{1}{ \vert 2^{k+3}\sqrt{n} Q \vert } \int_{2^{k+3}\sqrt{n} Q\setminus 2^{k+2}\sqrt{n} Q} \bigl\vert f_{2}(y_{2}) \bigr\vert \,dy_{2} \\ &\leq C\|b_{1}\|_{\dot{\wedge}_{\beta_{1}}}\|b_{2}\|_{\dot{\wedge }_{\beta_{2}}} M(f_{1}) (x)\sum_{k=1}^{\infty}2^{-k} \omega\bigl(2^{-k}\bigr)\frac {1}{ \vert 2^{k+3}\sqrt{n} Q \vert } \\ &\quad {}\times\int_{2^{k+3}\sqrt{n} Q\setminus 2^{k+2}\sqrt{n} Q} \bigl\vert f_{2}(y_{2}) \bigr\vert \,dy_{2} \\ &\leq C\|b_{1}\|_{\dot{\wedge}_{\beta_{1}}}\|b_{2}\|_{\dot{\wedge }_{\beta_{2}}} M(f_{1}) (x) M(f_{2}) (x). \end{aligned}$$

Similarly,

$$ N_{24} \leq C\|b_{1}\|_{\dot{\wedge}_{\beta_{1}}}\|b_{2} \|_{\dot{\wedge }_{\beta_{2}}} M(f_{1}) (x) M(f_{2}) (x). $$

For $y_{1},y_{2}\in(Q^{*})^{c}$, $|y_{1}-x_{Q}|\sim|y_{1}-z|$, and $|y_{2}-x_{Q}|\sim |y_{2}-z|$, by Minkowski’s inequality and the smooth condition (1.2) we get

$$\begin{aligned} N_{25}&\leq\frac{C\|b_{2}\|_{\dot{\wedge}_{\beta_{2}}}}{ \vert Q \vert ^{1+\beta _{1}/ n}} \int_{Q} \int_{(\mathbb{R}^{n})^{2}} \bigl\vert \bigl(b_{1}(y_{1})- \lambda_{1}\bigr) \bigr\vert \\ &\quad {}\times \biggl( \int_{0}^{\infty}\bigl\vert K_{t}(z,y_{1},y_{2})- K_{t}(x_{Q},y_{1},y_{2}) \bigr\vert ^{2}\frac{dt}{t} \biggr)^{\frac{1}{2}} \bigl\vert f_{1}^{\infty}(y_{1})f_{2}^{\infty}(y_{2}) \bigr\vert \,dy_{1}\,dy_{2} \,dz \\ &\leq\frac{C\|b_{1}\|_{\dot{\wedge}_{\beta_{1}}}\|b_{2}\|_{\dot{\wedge }_{\beta_{2}}}}{ \vert Q \vert ^{1+\beta_{1}/ n}} \int_{Q} \int_{(\mathbb {R}^{n})^{2}}\frac{ \vert y_{1}-x_{Q} \vert ^{\beta_{1}}}{( \vert z-y_{1} \vert + \vert z-y_{2} \vert )^{2n}} \\ &\quad {}\times \omega\biggl(\frac{ \vert z-x_{Q} \vert }{ \vert z-y_{1} \vert + \vert z-y_{2} \vert }\biggr) \bigl\vert f_{1}^{\infty}(y_{1})f_{2}^{\infty}(y_{2}) \bigr\vert \,dy_{1}\,dy_{2} \,dz \\ &\leq\frac{C\|b_{1}\|_{\dot{\wedge}_{\beta_{1}}}\|b_{2}\|_{\dot{\wedge }_{\beta_{2}}}}{ \vert Q \vert ^{1+\beta_{1}/ n}} \int_{Q} \int_{(\mathbb {R}^{n})^{2}}\frac{ \vert y_{1}-x_{Q} \vert ^{\beta_{1}} \vert f_{1}^{0}(y_{1})f_{2}^{\infty}(y_{2}) \vert }{( \vert z-y_{1} \vert + \vert z-y_{2} \vert )^{2n}} \\ &\quad {}\times \omega\biggl(\frac{ \vert z-x_{Q} \vert }{ \vert z-y_{1} \vert + \vert z-y_{2} \vert }\biggr)\,dy_{1} \,dy_{2} \,dz \\ &\leq\frac{C}{ \vert Q \vert ^{1+\beta_{1}/ n}}\|b_{1}\|_{\dot{\wedge}_{\beta _{1}}}\|b_{2} \|_{\dot{\wedge}_{\beta_{2}}} \int_{Q} \int_{((Q^{*})^{c})^{2} }\frac{ \vert f_{1}(y_{1}) \vert \vert f_{2}(y_{2}) \vert }{ \vert y_{1}-x_{Q} \vert ^{2n-\beta_{1}}} \omega \biggl(\frac{ \vert z-x_{Q} \vert }{ \vert z-y_{1} \vert } \biggr)\,dy_{1} \,dy_{2}\,dz \\ &\leq\frac{C}{ \vert Q \vert ^{1+\beta_{1}/ n}}\|b_{1}\|_{\dot{\wedge}_{\beta _{1}}}\|b_{2} \|_{\dot{\wedge}_{\beta_{2}}} \\ &\quad {}\times\int_{Q} \sum_{k=1}^{\infty}\int _{2^{k+3}\sqrt{n} Q\setminus2^{k+2}\sqrt{n} Q}\frac { \vert f_{1}(y_{1}) \vert \vert f_{2}(y_{2}) \vert }{ \vert y_{1}-x_{Q} \vert ^{2n-\beta_{1}}} \omega\biggl(\frac { \vert z-x_{Q} \vert }{ \vert z-y_{1} \vert } \biggr) \,dy_{1}\,dy_{2}\,dz \\ &\leq C\|b_{1}\|_{\dot{\wedge}_{\beta_{1}}}\|b_{2}\|_{\dot{\wedge }_{\beta_{2}}} \sum_{k=1}^{\infty}\frac{2^{k\beta_{1}}\omega (2^{-k})}{ \vert 2^{k+3}\sqrt{n} Q \vert ^{2}} \\ &\quad {}\times\int_{2^{k+3}\sqrt{n} Q\setminus 2^{k+2}\sqrt{n} Q} \bigl\vert f_{1}(y_{1}) \bigr\vert \,dy_{1} \int_{2^{k+3}\sqrt{n} Q\setminus2^{k+2}\sqrt{n} Q} \bigl\vert f_{2}(y_{2}) \bigr\vert \,dy_{2} \\ &\leq C\|b_{1}\|_{\dot{\wedge}_{\beta_{1}}}\|b_{2}\|_{\dot{\wedge }_{\beta_{2}}} M(f_{1}) (x) M(f_{2}) (x). \end{aligned}$$

Combining the estimates for $N_{21}$, $N_{22}$, $N_{23}$, $N_{24}$, $N_{25}$, we get

$$ N_{2} \leq C\|b_{1}\|_{\dot{\wedge}_{\beta_{1}}}\|b_{2} \|_{\dot{\wedge }_{\beta_{2}}}\bigl\{ M_{r}\bigl(T(f_{1},f_{2}) \bigr) (x)+M_{q_{1}}(f_{1}) (x)M_{q_{2}}(f_{2}) (x)+ M(f_{1}) (x) M(f_{2}) (x)\bigr\} . $$

Similarly, we have

$$ N_{3} \leq C\|b_{1}\|_{\dot{\wedge}_{\beta_{1}}}\|b_{2} \|_{\dot{\wedge }_{\beta_{2}}}\bigl\{ M_{r}\bigl(T(f_{1},f_{2}) \bigr) (x)+M_{q_{1}}(f_{1}) (x)M_{q_{2}}(f_{2}) (x)+ M(f_{1}) (x) M(f_{2}) (x)\bigr\} $$

and

$$ N_{4} \leq C\|b_{1}\|_{\dot{\wedge}_{\beta_{1}}}\|b_{2} \|_{\dot{\wedge }_{\beta_{2}}}\bigl\{ M_{r}\bigl(T(f_{1},f_{2}) \bigr) (x)+M_{q_{1}}(f_{1}) (x)M_{q_{2}}(f_{2}) (x)+ M(f_{1}) (x) M(f_{2}) (x)\bigr\} . $$

Thus we deduce that

$$\begin{aligned} &\frac{1}{|Q|^{1+\beta/ n}} \int_{Q} \bigl\vert T_{\Pi\vec {b}}(\vec{f}) (z)- \bigl(T_{\Pi\vec {b}}(\vec{f})\bigr)_{Q} \bigr\vert \,dz \\ &\quad \leq C\|b_{1}\|_{\dot{\wedge}_{\beta_{1}}}\|b_{2} \|_{\dot{\wedge }_{\beta_{2}}}\bigl\{ M_{r}\bigl(T(f_{1},f_{2}) \bigr) (x)+M_{q_{1}}(f_{1}) (x)M_{q_{2}}(f_{2}) (x)+ M(f_{1}) (x) M(f_{2}) (x)\bigr\} . \end{aligned}$$

By the Hölder inequality, Lemma 2.3, and the normal inequalities for the maximal operators, we arrive at

$$\begin{aligned} & \bigl\Vert T_{\Pi\vec {b}}(\vec{f}) \bigr\Vert _{\dot{F}_{p}^{\beta,\infty}} \\ &\quad \approx \biggl\Vert \sup_{Q}\frac{1}{|Q|^{1+\beta/ n}} \int_{Q} \bigl|T_{\Pi\vec {b}}(\vec{f}) (z)- \bigl(T_{\Pi\vec {b}}(\vec{f})\bigr)_{Q}\bigr|\,dz \biggr\Vert _{L^{p}} \\ &\quad \leq C \Vert b_{1} \Vert _{\dot{\wedge}_{\beta_{1}}} \Vert b_{2} \Vert _{\dot{\wedge }_{\beta_{2}}}\bigl\{ \bigl\Vert M_{r} \bigl(T(f_{1},f_{2})\bigr) \bigr\Vert _{L^{p}}+ \bigl\Vert M_{q_{1}}(f_{1})M_{q_{2}}(f_{2}) \bigr\Vert _{L^{p}}+ \bigl\Vert M(f_{1}) M(f_{2}) \bigr\Vert _{L^{p}}\bigr\} \\ &\quad \leq C \Vert b_{1} \Vert _{\dot{\wedge}_{\beta_{1}}} \Vert b_{2} \Vert _{\dot{\wedge }_{\beta_{2}}}\bigl\{ \bigl\Vert T(f_{1},f_{2}) \bigr\Vert _{L^{p}}+ \bigl\Vert M_{q_{1}}(f_{1}) \bigr\Vert _{L^{p_{1}}} \bigl\Vert M_{q_{2}}(f_{2}) \bigr\Vert _{L^{p_{2}}} \\ &\qquad {}+ \bigl\Vert M(f_{1}) \bigr\Vert _{L^{p_{1}}} \bigl\Vert M(f_{2}) \bigr\Vert _{L^{p_{2}}} \bigr\} \\ &\quad \leq \Vert b_{1} \Vert _{\dot{\wedge}_{\beta_{1}}} \Vert b_{2} \Vert _{\dot{\wedge}_{\beta _{2}}} \Vert f_{1} \Vert _{L^{p_{1}}} \Vert f_{2} \Vert _{L^{p_{2}}}, \end{aligned}$$

where we have used that facts $1 < r < p$, $1 < q_{1} < p_{1}$, and $1 < q_{2} < p_{2}$. This finishes the proof of Theorem 1.3. □

## Conclusions

In this paper,we studied the boundedness properties of the commutator generated by a multilinear square function and Lipschitz functions with kernel satisfying Dini-type condition. We showed that such commutators are bounded from product Lebesgue spaces into the Lebesgue spaces, Lipschitz spaces, and Triebel–Lizorkin spaces.
